# A Human Model of Oligodendrocyte Development Shows MCL‐1 Influences Oligodendrocyte Morphogenesis

**DOI:** 10.1002/glia.70128

**Published:** 2025-12-19

**Authors:** Melanie Gil, Marina R. Hanna, Vivian Gama

**Affiliations:** ^1^ Department of Cell and Developmental Biology Vanderbilt University Nashville Tennessee USA; ^2^ Neuroscience Graduate Program Vanderbilt University Nashville Tennessee USA; ^3^ Vanderbilt Brain Institute Vanderbilt University Nashville Tennessee USA; ^4^ Vanderbilt Center for Stem Cell Biology Vanderbilt University Nashville Tennessee USA

**Keywords:** fatty acid oxidation, hESCs, iPSCs, MCL‐1, mitochondria, oligodendrocytes

## Abstract

Oligodendrocytes are the myelinating cells of the central nervous system. Regulation of the early stages of oligodendrocyte development is critical to the function of the cell. Specifically, myelin sheath formation is an energetically demanding event that requires precision, as alterations may lead to dysmyelination. Fatty acid β‐oxidation has been shown to be critical for the function of oligodendrocytes. We previously showed that myeloid cell leukemia‐1 (MCL‐1), a well‐characterized anti‐apoptotic protein, is required for the development of murine oligodendrocytes in vivo. Further, MCL‐1 regulates long‐chain fatty acid β‐oxidation in cancer cells through its interaction with Acyl‐CoA synthetase long‐chain family member 1 (ACSL1), an enzyme responsible for the conversion of free long‐chain fatty acids into fatty acyl‐CoA esters. Here, we introduce an in vitro system to isolate human stem cell‐derived oligodendrocyte progenitor cells (OPCs) and investigate the involvement of MCL‐1 during human oligodendrocyte development. Using this system, we pharmacologically inhibited MCL‐1 in OPCs to investigate its non‐apoptotic function at this developmental stage. We also used a motor neuron‐oligodendrocyte co‐culture system to examine the downstream effects of MCL‐1 at later developmental stages when oligodendrocytes begin to contact axons and generate myelin. We demonstrate that the mitochondrial network changes in human oligodendrocyte development resemble those reported in mouse tissue. Our findings point to MCL‐1 as a critical factor essential for proper oligodendrocyte morphogenesis.

## Graphical abstract

A unified model of oligodendrocyte differentiation from human embryonic stem cells revealed that MCL‐1 is critical for regulating the expression of oligodendrocyte‐related genes and the morphogenesis of myelinating oligodendrocytes.

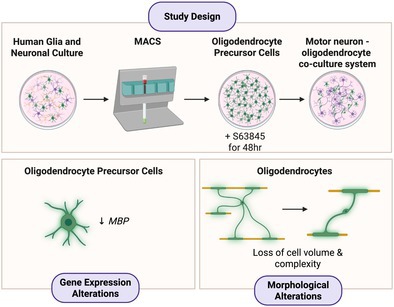

## 1 Introduction

Oligodendrogenesis, the production of myelin‐forming oligodendrocytes, is a fundamental process during brain development. This process is energetically demanding to efficiently form and maintain the myelin sheath. Additionally, oligodendrocytes provide metabolic support to the axons they myelinate, increasing their bioenergetic demands. Evidence from previous studies demonstrates that oligodendrocyte precursor cells (OPCs) have high oxidative phosphorylation (OXPHOS) rates before and during myelination, while myelinating oligodendrocytes have low OXPHOS and high glycolytic rates (Fünfschilling et al. 2012; Ziabreva et al. 2010). The mechanisms regulating this metabolic switch are not well understood. However, mitochondria undergo morphological remodeling during maturation in vivo, supporting a shift in metabolic demands (Bame and Hill 2024). The dependence on fatty acid oxidation (FAO) during this maturation stage is poorly understood in oligodendrocytes. Mitochondrial FAO generates acetyl‐CoA to support the tricarboxylic acid (TCA) cycle. Cells typically depend on FAO when glucose levels in the cell are low. Previous studies have shown that FAO is required for neural stem cell maintenance and proper neurogenesis (Knobloch et al. 2017). Fatty acid β‐oxidation in oligodendrocytes supports axonal energy metabolism when glucose is depleted, indicating that oligodendrocyte fatty acid metabolism can serve as an energy reserve for the bioenergetic needs of these cells (Asadollahi et al. 2024).

MCL‐1 is a well‐characterized anti‐apoptotic protein, known to inhibit mitochondrial‐mediated cell death. MCL‐1 has non‐apoptotic functions that are critical for postembryonic development (Brinkmann et al. 2025). We have previously shown that MCL‐1 regulates mitochondrial morphology and dynamics of human pluripotent stem cells as well as other differentiated cells such as cardiomyocytes (Rasmussen et al. 2020; Rasmussen et al. 2018). This non‐apoptotic function of MCL‐1 is critical for maintaining the bioenergetic needs of cells during differentiation. MCL‐1 also promotes long‐chain fatty acid β‐oxidation by interacting with acyl‐CoA synthetase long‐chain family member 1 (ACSL1), an enzyme that converts long‐chain fatty acids into acyl‐CoAs (Wright et al. 2024). This interaction is disrupted by the MCL‐1 inhibitor and BH‐3 mimetic, S63845 (Wright et al. 2024; Kotschy et al. 2016). We previously showed that knocking out *Mcl1* in mouse neural progenitor cells leads to a decrease in myelinating oligodendrocytes (Cleveland et al. 2021). Together, these findings suggest a potential link between oligodendrocyte function and the non‐apoptotic role of MCL‐1.

To investigate the mechanisms that modulate the development of human oligodendrocytes, we unified an in vitro system based on previous methods to isolate human embryonic stem cell (hESC)‐derived OPCs that undergo maturation when co‐cultured with motor neurons (Gil and Gama 2024; Ziller et al. 2018; Douvaras and Fossati 2015; García‐León et al. 2018). This system allows us to investigate downstream effects on mitochondrial morphology, gene expression of FAO enzymes, and expression of oligodendrocyte‐associated genes following treatment. Additionally, using the co‐culture system, we found that pharmacological inhibition of MCL‐1 at the OPC stage leads to morphological changes in oligodendrocytes expressing myelin basic protein (MBP) and alterations to their mitochondrial morphology. This is the first report demonstrating remodeling of mitochondrial morphology during the development of human oligodendrocytes and points to an essential role for MCL‐1 at initial stages of oligodendrocyte development.

## 2 Results

### 2.1 Generation of the Oligodendrocyte Lineage From Human Stem Cells

**FIGURE 1:**
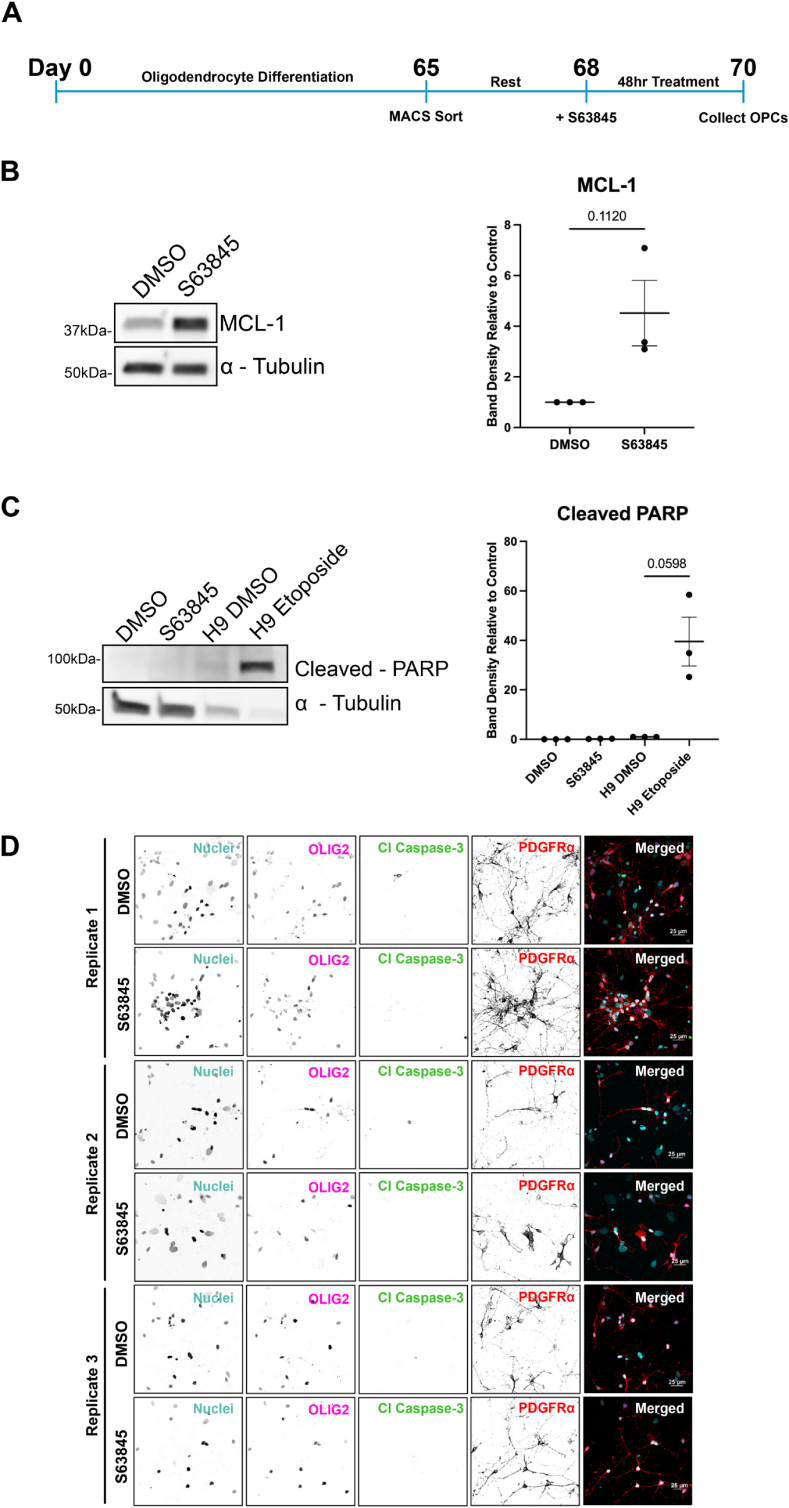
In vitro human oligodendrocyte system reveals the non‐apoptotic effect of MCL‐1 inhibition. (A) Timeline of oligodendrocyte differentiation and treatment with S63845. At day 65 of differentiation, cells undergo cell sorting using the MACS cell separation system. Following a rest period, cells were treated with DMSO (vehicle) or S63845 for 48 h. At the 48‐h timepoint, cells were collected. (B) Western blot of MCL‐1 levels in OPCs following DMSO (vehicle) and S63845 treatment (left panel) and band density quantification (right panel). Each dot on graphs represents a biological replicate (*n* = 3), analyzed by student's *t*‐test, and error bars represent mean ± SEM. (C) Western blot of cleaved PARP following S63845 treatment in OPCs and etoposide treatment in H9‐line human embryonic stem cells, respectively (left panel) and band density quantification (right panel). Both treatments used DMSO as vehicle control. Each dot on graphs represents a biological replicate (*n* = 3), analyzed by student's *t*‐test, error bars represent mean ± SEM. (D) Representative spinning disk confocal maximum intensity projections from each biological replicate of immunofluorescent staining for nuclei (cyan), OLIG2 (magenta), Cleaved Caspase‐3 (Cl Caspase‐3) (green), and platelet‐derived growth factor receptor alpha (PDGFRα) (red) (scale bar = 25 μm).

Several protocols have been described for the differentiation of human stem cells into oligodendrocytes (Douvaras and Fossati 2015; Hu et al. 2009; Izrael et al. 2007; Nistor et al. 2005; García‐León et al. 2020; Biswas et al. 2019). Using these previous reports, we optimized a single protocol for the generation of myelinating oligodendrocytes from stem cells that can be used to manipulate and investigate oligodendrocyte lineage cells at the various stages of development (Gil and Gama 2024). A mixed culture consisting of OPCs, neurons, and astrocytes was generated using a series of small molecule and growth‐factor‐defined media (Douvaras and Fossati 2015). To purify the population of OPCs, we used the MACS cell separation system with Anti‐A2B5 MicroBeads at 65 days in vitro (DIV) (Figure 1A). OLIG2‐positive cells were, on average, 72% of the cell population following the cell sort (Figure S1A,B). GFAP‐positive cells were present in this population of cells (Figure S1C). To further elucidate the non‐apoptotic function of MCL‐1 during the development of oligodendrocytes, we treated isolated OPCs with S63845 for 48 h. Previous studies outlining the mechanism of action of S63845 found that inhibition of MCL‐1 leads to increased protein stability, consequently increasing protein levels (Kotschy et al. 2016). Following the treatment of OPCs, protein levels of MCL‐1 increased as anticipated, confirming the effectiveness of pharmacological MCL‐1 inhibition (Figure 1B).

### 2.2 MCL‐1 Inhibition Does Not Lead to Cell Death in Oligodendrocyte Precursor Cells

MCL‐1 is a highly regulated protein that inhibits cell death by interacting with pro‐apoptotic proteins through its BH3 domain (Shamas‐Din et al. 2013; Sancho et al. 2022). The anti‐apoptotic function of MCL‐1 has a specific temporal requirement during early neurogenesis, and it has been shown that it is necessary for the survival of neural progenitor cells in the developing mouse brain (Fogarty et al. 2019; Arbour et al. 2008). Recent findings show that the apoptotic function of MCL‐1 is critical for embryonic development, and the non‐apoptotic function of MCL‐1 is needed for postembryonic development (Brinkmann et al. 2025). The role of MCL‐1 during oligodendrogenesis is not known. Our findings demonstrate that MCL‐1 is dispensable for the survival of human OPCs. Following treatment of OPCs with S63845 for a 48‐h period, protein levels of cleaved‐PARP, an indicator of caspase activation, were not altered (Figure 1C). As a positive control to validate the protein levels of cleaved‐PARP, we treated hESCs (H9 line) with etoposide, a DNA‐damaging agent that leads to cell death. Following treatment with etoposide, cleaved‐PARP levels were increased, indicating caspase‐mediated apoptosis. To complement these studies, we also treated OPCs with S63845 and performed immunofluorescence for cleaved caspase‐3, which was not increased after treatments with the inhibitor (Figure 1D). To validate cleaved caspase‐3 immunostaining, we treated hESC‐derived neural progenitor cells with etoposide, a DNA‐damaging agent. Etoposide exposure led to cell death as demonstrated by cleaved caspase‐3 expression, which was absent in the DMSO‐treated group (Figure S2A). The absence of cell death following pharmacological inhibition of MCL‐1 indicates that the findings reported in this study are independent of a cell death phenotype.

### 2.3 MCL‐1 Inhibition Does Not Alter Mitochondrial Morphology in Oligodendrocyte Precursor Cells

Mitochondria are dynamic organelles constantly undergoing coordinated fusion and fission events needed to maintain mitochondrial function and cellular homeostasis (Detmer and Chan 2007). Mitochondrial fission events are primarily mediated by dynamin‐related protein 1 (DRP1), while fusion events are mediated by mitofusin 1/2 (MFN1/2) and optic atrophy 1 (OPA1) (Liesa et al. 2009). We and others have shown that alterations to DRP1 can lead to detrimental changes in mitochondrial function and, subsequently, neuronal and glial function (Robertson et al. 2023; Baum et al. 2024; Salazar et al., n.d.). Thus, it is critical to understand the tight regulation of these processes during neuronal and glial development as they have broader contributions to cellular function. The modifications of mitochondrial dynamics and ultrastructure determine the metabolic state of the cell and its adaptation to a new state (Benard et al. 2007; Picard et al. 2013; Soares et al. 2024).

**FIGURE 2:**
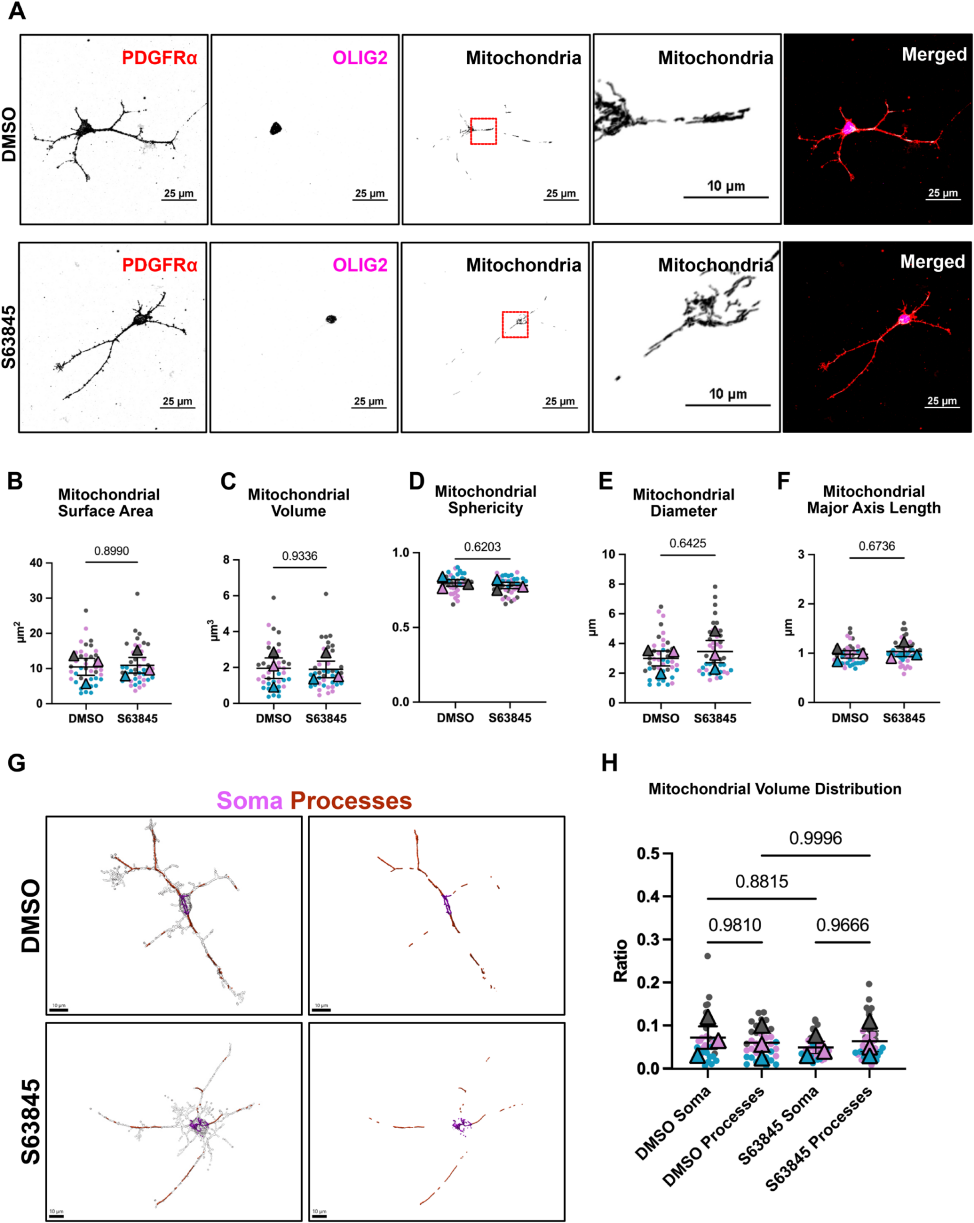
Pharmacological inhibition of MCL‐1 does not cause alterations to mitochondrial morphology in oligodendrocyte progenitor cells. (A) Representative spinning disk confocal maximum intensity projections of immunofluorescent staining for platelet‐derived growth factor receptor alpha (PDGFRα) (red), oligodendrocyte transcription factor 2 (OLIG2) (magenta), and mitochondria (white) in DMSO (vehicle) and S63845 treated OPCs (scale bar = 25 μm) zoom in of mitochondria at the nucleus (scale bar = 10 μm). (B–F) Quantification of mitochondrial surface area, volume, sphericity, diameter, and major axis length. Each color represents a biological replicate (*n* = 3), each dot represents a cell (10–15 per *n*), each triangle represents the mean of the biological replicate, analyzed by student's *t*‐test, and error bars represent mean ± SEM. (G) 3D reconstructions of OPCs and their mitochondrial network treated with DMSO (vehicle) and S63845. Mitochondria in magenta are in the cell's soma and mitochondria in red are in the cell's processes (scale bar = 10 μm). (H) Distribution of mitochondria in OPCs. Ratio calculated by diving the volume of the mitochondria in the soma (shown in magenta in G) or processes (shown in red in G) by the total volume of the OPC. Each color represents a biological replicate (*n* = 3), each dot represents a cell (10–15 per *n*), each triangle represents mean of biological replicate, analyzed by one‐way ANOVA, error bars represent mean ± SEM. Conditions were blinded to experimenter for 3D reconstructions.

We have previously shown that inhibition of MCL‐1 leads to mitochondrial elongation in hESCs and fragmentation in cardiomyocytes derived from human induced pluripotent stem cells (iPSCs) through the potential interplay with DRP1 and OPA1 (Rasmussen et al. 2018; Rasmussen et al. 2020). These findings indicate that MCL‐1 helps to maintain mitochondrial morphology, which is essential for energy homeostasis. The role of MCL‐1 in mitochondrial function during oligodendrocyte development is largely unknown. We treated isolated OPCs with S63845 for 48 h and did not see significant morphological changes to the mitochondria. We used oligodendrocyte transcription factor 2 (OLIG2) and platelet‐derived growth factor receptor alpha (PDGFRα) as markers to identify OPCs following treatment (Figure 2A) (Hall et al. 1996; Lu et al. 2000). This co‐staining method allowed for visualization of the mitochondrial network within the entire cell. There were no significant changes to the mitochondrial surface area, volume, sphericity, diameter, or major axis length in response to MCL‐1 inhibition (Figure 2B–F).

The mitochondria in OPCs appear elongated, and the mitochondrial network expands throughout the soma and processes of the cell. The mitochondria appear to wrap around the nucleus and continue to reach into the processes where they may be needed to meet the energetic demands of the growing cell. Bame and Hill (2024) reported an in vivo mouse system to characterize mitochondrial network reorganization throughout the development of oligodendrocytes. The characterization of the mitochondrial morphology in our in vitro model recapitulates their findings in the OPC stage. To compare the two model systems, we used a similar analytic workflow that allows for comparison of the mitochondrial volume in the soma and processes of the OPC. To our knowledge, this is the first report of mitochondrial morphology remodeling in human OPCs in an in vitro system. After generating 3D reconstructions of OPCs using Imaris image analysis software, we developed a ratio to quantify the volume of mitochondria within the soma or processes normalized to the total volume of the OPC (Figure 2G). We found no significant differences in the mitochondrial distribution between the soma and the processes (Figure 2H). Inhibition of MCL‐1 did not modify the distribution of mitochondria within the cell. Additionally, there are no significant differences in the volume of PDGFRα positive cells (Figure S3). Thus, the pharmacological inhibition of MCL‐1 does not affect the distribution of mitochondria within OPCs.

### 2.4 MCL‐1 Inhibition in Human Oligodendrocyte Precursor Cells Does Not Impact the Fatty Acid β‐Oxidation Pathway

**FIGURE 3:**
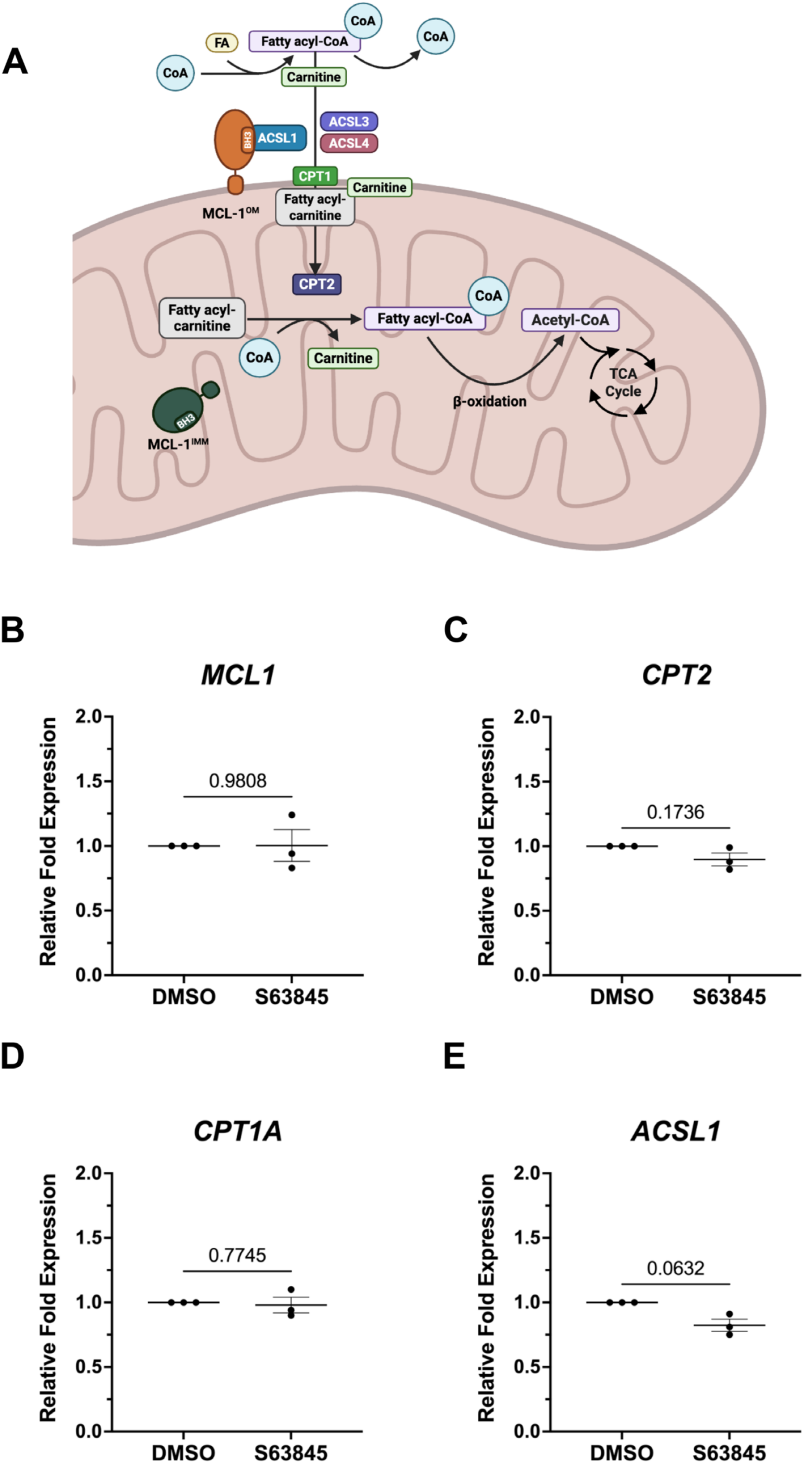
Pharmacological inhibition of MCL‐1 leads to decreased gene expression of *ACSL1*. (A) Schematic outlining activation of fatty acids (FA) and subsequent transport into the mitochondrial matrix for oxidation. MCL‐1 at the outer mitochondrial membrane (MCL‐1^OM^) may associate with ACSL1 promoting its function. ACSL1 catalyzes the conversion of long‐chain fatty acids to their activated form, fatty acyl‐CoA, which can cross the outer mitochondrial membrane and enter the intermembrane space. To be transported into the mitochondrial matrix, fatty acyl‐CoA must be converted to fatty acyl‐carnitine. Carnitine palmitoyltransferase 1 (CPT1) catalyzes the transfer of the acyl group of a fatty acyl‐CoA from coenzyme A to carnitine, allowing fatty acyl‐carnitine to enter the matrix. Once inside the mitochondria matrix, carnitine palmitoyltransferase 2 (CPT2) converts fatty acyl‐carnitine back to fatty acyl‐CoA. β‐oxidation breaks down fatty acyl‐CoA to acetyl‐CoA. Acetyl‐CoA molecules then enter the tricarboxylic acid (TCA) cycle to generate ATP. (B–E) qPCR relative fold expression of *MCL1*, *ACSL*, *CPT1A*, and *CPT2*. Each dot on graphs represents a biological replicate (*n* = 3) consisting of the average of two technical replicates, analyzed by student's *t*‐test; error bars represent mean ± SEM.

OPCs are critical during oligodendrogenesis as they proliferate and simultaneously integrate into the developing nervous system (Baum et al. 2024). At this stage, they have high energetic demands as they extend their processes to search for axons and prepare to generate myelin sheaths, which are lipid‐rich structures (Barres and Raff 1993). OPCs rely on OXPHOS and switch to glycolysis as they mature into myelinating oligodendrocytes (Fünfschilling et al. 2012; Ziabreva et al. 2010). Previous findings established that oligodendrocytes depend on fatty acid β‐oxidation in low glucose conditions (Asadollahi et al. 2024). Thus, ATP produced from the oxidation of fatty acids in the mitochondria is sufficient to maintain the function of the oligodendrocyte when glycolysis is not available. However, the role of FAO during this developmental switch is not well understood. MCL‐1 has been reported to regulate long‐chain fatty acid β‐oxidation by its interaction with ACSL1 (Wright et al. 2024). Activation of fatty acids by ACSL1 triggers a cascade of events regulated by CPT1 and CPT2, two key enzymes that work together to transport fatty acids into the mitochondria (Wang et al. 2021). Once inside the mitochondria, fatty acyl‐CoA undergoes β‐oxidation to generate acetyl‐CoA for the TCA cycle (Houten and Wanders 2010; Talley and Mohiuddin 2024; Koltai et al. 2021) (Figure 3A). It has been previously reported that FAO genes are downregulated following the genetic deletion of *Mcl‐1* in mouse B‐cell lymphoblastic leukemia cells and liver tissue (Prew et al. 2022). To understand if this regulatory mechanism is conserved in OPCs, we examined changes in gene expression of *MCL1*, *ACSL1*, *CPT1*, and *CPT2* following inhibition of MCL‐1. Gene expression of *MCL1*, *CPT1A*, and *CPT2* was not altered. While *ACSL1* levels were lower in MCL‐1 inhibitor‐treated cells compared to untreated cells, this difference was not statistically significant (*p* = 0.0632) (Figure 3B–E). These results indicate that, unlike results in other systems, MCL‐1 has only a marginal effect on fatty acid metabolism in OPCs (Wright et al. 2024).

### 2.5 Targeting Enzymes of the Mitochondrial Fatty Acid Oxidation Pathway in the Absence of Glucose in OPCs Causes Dysregulation in Lipid Storage

**FIGURE 4:**
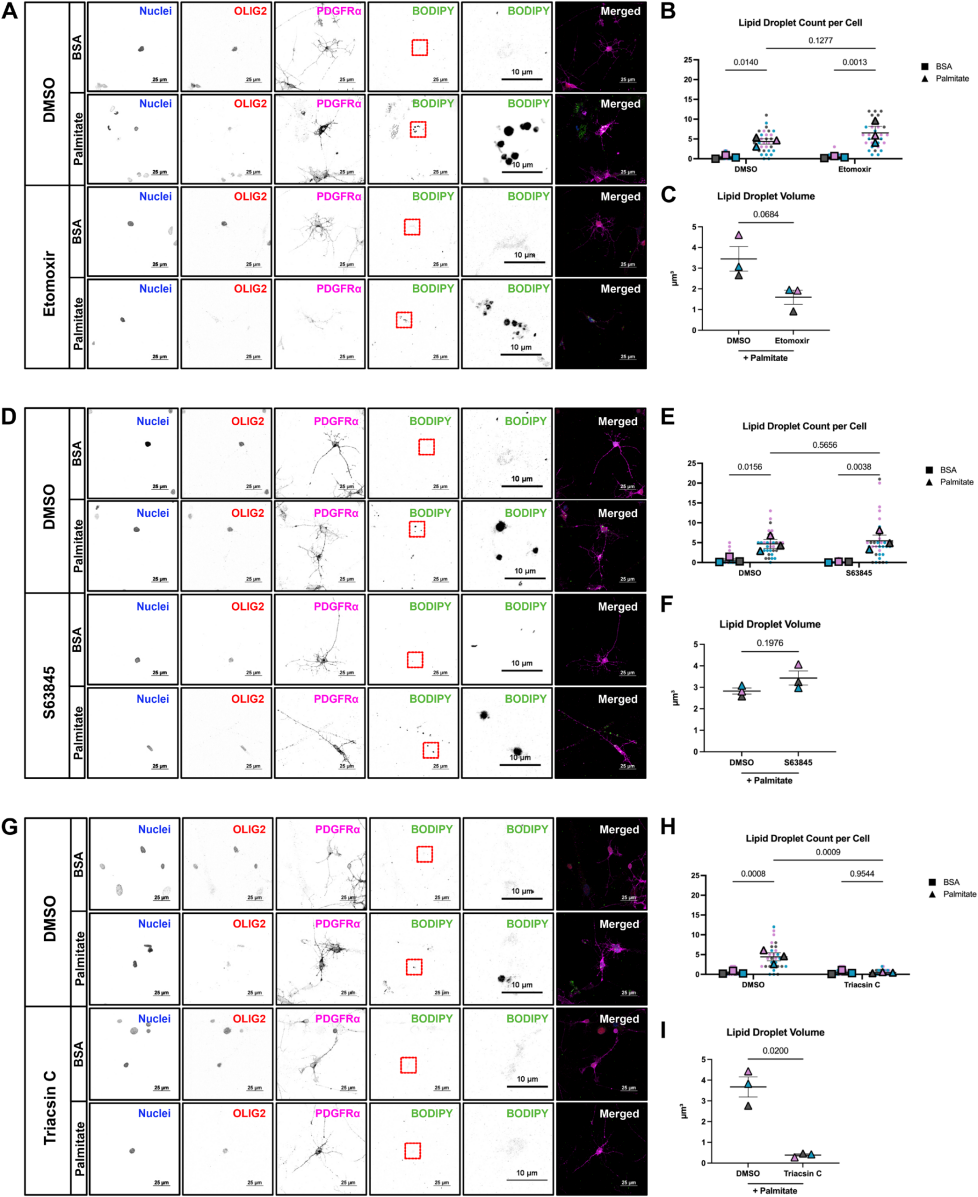
Targeting enzymes of the mitochondrial fatty acid oxidation pathway in a glucose‐depleted state. (A) Representative spinning disk confocal maximum intensity projections of immunofluorescent staining for nuclei (blue), oligodendrocyte transcription factor 2 (OLIG2) (red), platelet‐derived growth factor receptor alpha (PDGFRα) (magenta), and BODIPY (green) in DMSO (vehicle) and etomoxir treated OPCs that were supplemented with BSA‐palmitate saturated fatty acid complex or BSA (vehicle) (scale bar = 25 μm). Zoom‐in of lipid droplets (scale bar = 10 μm). (B) Quantification of lipid droplets per cell in DMSO and etomoxir treated groups supplemented with BSA or BSA‐palmitate. Each color represents a biological replicate (*n* = 3), each dot represents a cell (8–12 per *n*), each square or triangle represents the mean of the biological replicate, analyzed using an ordinary two‐way ANOVA, error bars represent mean ± SEM. (C) Quantification of lipid droplet volume in DMSO and etomoxir treated groups supplemented with BSA or BSA‐palmitate. Each color represents a biological replicate (*n* = 3), each triangle represents the mean of the biological replicate, analyzed by student's *t*‐test; error bars represent mean ± SEM. (D) Representative spinning disk confocal maximum intensity projections of immunofluorescent staining for nuclei (blue), oligodendrocyte transcription factor 2 (OLIG2) (red), platelet‐derived growth factor receptor alpha (PDGFRα) (magenta), and BODIPY (green) in DMSO (vehicle) and S63845 treated OPCs that were supplemented with BSA‐palmitate saturated fatty acid complex or BSA (vehicle) (scale bar = 25 μm). Zoom‐in of lipid droplets (scale bar = 10 μm). (E) Quantification of lipid droplets per cell in DMSO and S63845 treated groups supplemented with BSA or BSA‐palmitate. Each color represents a biological replicate (*n* = 3), each dot represents a cell (8–12 per *n*), each square or triangle represents the mean of the biological replicate, analyzed using an ordinary two‐way ANOVA, error bars represent mean ± SEM. (F) Quantification of lipid droplet volume in DMSO and S63845 treated groups supplemented with BSA or BSA‐palmitate. Each color represents a biological replicate (*n* = 3), each triangle represents the mean of the biological replicate, analyzed by student's *t*‐test; error bars represent mean ± SEM. (G) Representative spinning disk confocal maximum intensity projections of immunofluorescent staining for nuclei (blue), oligodendrocyte transcription factor 2 (OLIG2) (red), platelet‐derived growth factor receptor alpha (PDGFRα) (magenta), and BODIPY (green) in DMSO (vehicle) and Triacsin C treated OPCs that were supplemented with BSA‐palmitate saturated fatty acid complex or BSA (vehicle) (scale bar = 25 μm). Zoom‐in of lipid droplets (scale bar = 10 μm). (H) Quantification of lipid droplets per cell in DMSO and Triacsin C treated groups supplemented with BSA or BSA‐palmitate. Each color represents a biological replicate (*n* = 3), each dot represents a cell (8–12 per *n*), each square or triangle represents the mean of the biological replicate, analyzed using an ordinary two‐way ANOVA, error bars represent mean ± SEM. (I) Quantification of lipid droplet volume in DMSO and Triacsin C treated groups supplemented with BSA or BSA‐palmitate. Each color represents a biological replicate (*n* = 3), each triangle represents the mean of the biological replicate, analyzed by student's *t*‐test; error bars represent mean ± SEM.

Lipid droplets are organelles that store energy in the form of neutral lipids and serve as nutrient reservoirs during starvation (Welte and Gould 2017). Pharmacological inhibition of CPT1 by etomoxir leads to lipid droplet accumulation, as long‐chain fatty acids, such as palmitate, cannot undergo oxidation. Thus, the accumulation of lipid droplets may serve as an indicator of alterations in FAO. We first inhibited FAO with etomoxir and tracked lipid droplet accumulation with BODIPY in the absence of glucose. We cultured OPCs in HBSS supplemented with BSA‐palmitate saturated fatty acid complex or BSA as a vehicle control for 24 h to force reliance on FAO. We also treated OPCs with etomoxir or DMSO as a vehicle control during the 24‐h period and tracked lipid droplet accumulation. An increase in lipid droplets in OPCs supplemented with palmitate in both DMSO and etomoxir‐treated groups was observed (Figure 4A). The number of lipid droplets was not altered by etomoxir treatment, but a decrease in volume was detected (Figure 4B,C). Whether lipid droplets are trafficked to the mitochondria for oxidation or if more time is needed in a glucose‐depleted state to observe alterations in the number of lipid droplets accumulated is not known.

Building on previous findings that MCL‐1 regulates mitochondrial FAO through its interaction with ACSL1, along with our observation that MCL‐1 inhibition induces a decrease in ACSL1 gene expression, we investigated lipid droplet homeostasis in OPCs by treating them with S63845 for 24 h using the previously described glucose depletion approach (Wright et al. 2024). As with etomoxir, there is an increase in lipid droplet number in groups supplemented with palmitate, but no difference between the DMSO and S63845 treated groups (Figure 4D,E). There are no changes in lipid droplet volume following MCL‐1 inhibition and no changes in immunofluorescence intensity of OLIG2 and PDGFRα (Figure 4F, Figure S4A,B). A decrease in immunofluorescence intensity of OLIG2 in etomoxir‐treated cells supplemented with palmitate indicates a possible alteration to the function of OPCs; however, there were no significant changes to immunofluorescence intensity of PDGFRα (Figure S4A,B). Disrupting the MCL‐1/ACSL1 interaction represses FAO (Wright et al. 2024), thus, we decided to target ACSL1 using Triacsin C, a pan‐ACSL inhibitor, to identify the alterations to lipid droplet accumulation. The ACSL family of enzymes converts saturated and unsaturated fatty acids into fatty acyl‐CoA esters, which can be used for fatty acid β‐oxidation or synthesis of phospholipids and triacylglycerols (Quan et al. 2021). Triacsin C targets ACSL1, ACSL3, and ACSL4 (Fujimoto et al. 2007; Kim et al. 2001). Following treatment with Triacsin C for 24 h using the glucose depletion approach, a decrease in the number of lipid droplets in OPCs supplemented with palmitate was detected, which was accompanied by a loss of volume (Figure 4G–I).

Although some variability, where there were no significant changes in immunofluorescence intensity of OLIG2 and PDGFRα following inhibition of ACSL1 (Figure S4E,F). The robust loss of lipid droplets following inhibition of ACSL1 may be attributed to the dual function of the enzymes in activating fatty acids for both oxidation and synthesis. To gain a better understanding of the cell state, we examined mitochondrial morphology following treatment of OPCs with Triacsin C for 16 h in standard culture conditions. We observed the dramatic fragmentation of the mitochondrial network and its remodeling into perinuclear clustering, indicative of a mitochondrial stress response and dysfunction of FAO (Al‐Mehdi et al. 2012; Agarwal and Ganesh 2020) (Figure S5A,B).

### 2.6 Inhibition of MCL‐1 in Human Oligodendrocyte Precursor Cells Leads to Decreased Expression of *MBP*

**FIGURE 5:**
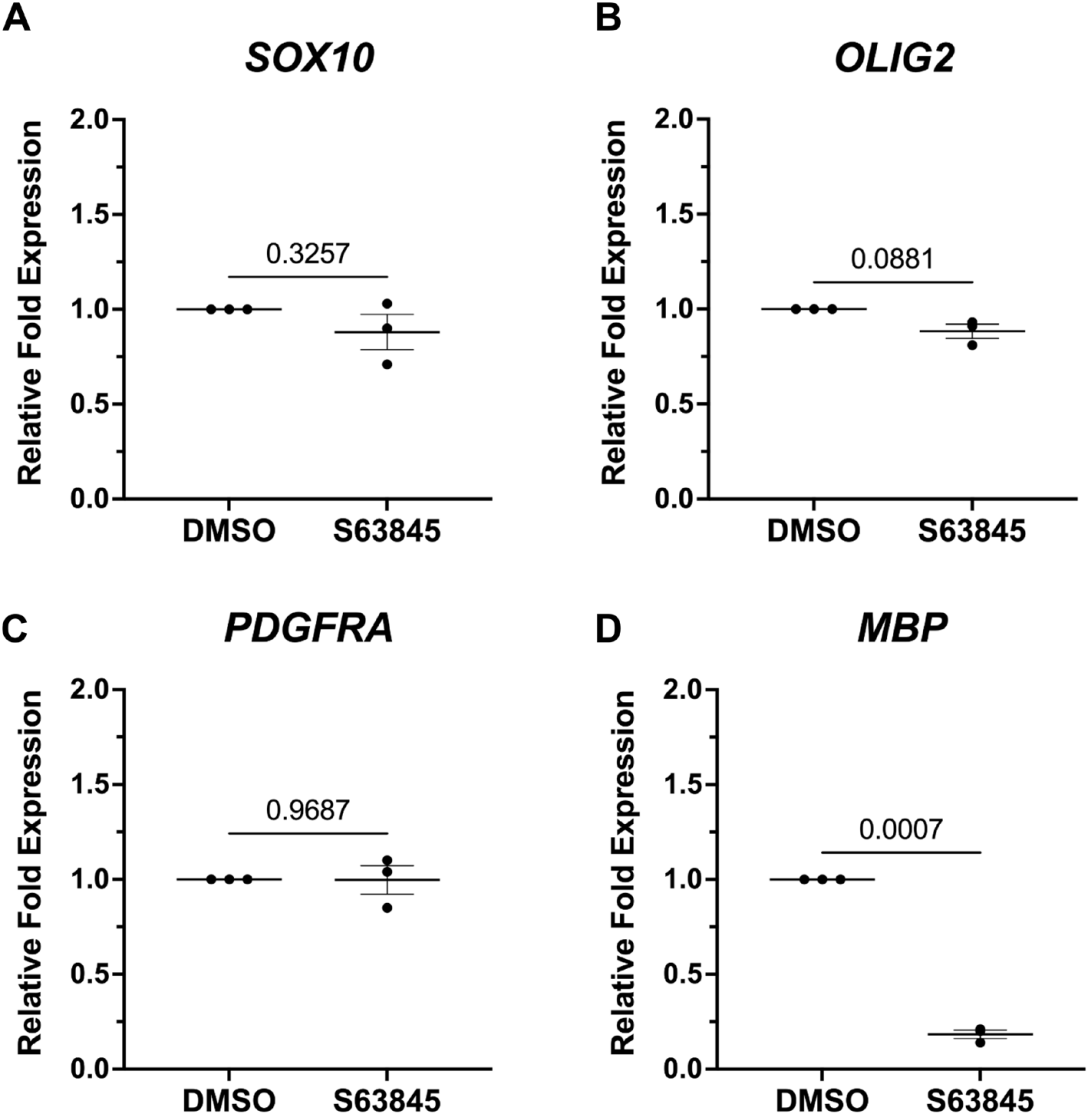
Pharmacological inhibition of MCL‐1 leads to decreased gene expression of *OLIG2* and *MBP*. (A–D) qPCR relative fold expression of *SOX10*, *OLIG2*, *PDGFRA*, and *MBP*. Each dot on graphs represents a biological replicate (*n* = 3) consisting of the average of two technical replicates, analyzed by student's *t*‐test, error bars represent mean ± SEM.

SRY‐box transcription factor 10 (SOX10) and OLIG2 are both key transcription factors that are involved in the development and maturation of oligodendrocytes (Rowitch 2004; Takebayashi et al. 2002; Takada et al. 2010). OLIG2 is expressed early in ventral progenitor cells, and SOX10 is present in the pre‐OPC to myelinating oligodendrocyte stages (Sock and Wegner 2021; Ravanelli and Appel 2015). SOX10 and OLIG2 modulate each other to control oligodendrocyte differentiation (Yue et al. 2006; Mei et al. 2013; Liu et al. 2007; Küspert et al. 2011; Stolt et al. 2002). The expression of both OLIG2 and SOX10 at the OPC stage ensures that transcriptional regulatory networks are functional in maintaining oligodendrocyte lineage identity (Sock and Wegner 2021). MBP is the most abundant protein in compact myelin (Han et al. 2013). It is responsible for interacting with lipids to maintain the structure of the myelin sheath (Boggs 2006; Raasakka et al. 2017; Weil et al. 2016). Loss of MBP leads to severe dysmyelination in the central nervous system (Jean Harry and Toews 1998; Privat et al. 1979). Previous findings from our group have revealed that the conditional deletion of *Mcl‐1* in neural progenitor cells leads to the loss of SOX10 and MBP in the developing mouse brain (Cleveland et al. 2021). We measured gene expression of *SOX10*, *OLIG2*, *PDGFRA*, and *MBP* following pharmacological inhibition of MCL‐1 in OPCs. Gene expression of *MBP* was significantly downregulated; however, expression of *OLIG2*, *SOX10*, and *PDGFRA* was maintained (Figure 5A–D). Accordingly, there were no changes in SOX10 or OLIG2 protein levels following treatment (Figure S6A,B).

### 2.7 Inhibition of MCL‐1 at Early Stages of Differentiation Decreases the Mitochondrial Major Axis Length in Oligodendrocytes

**FIGURE 6:**
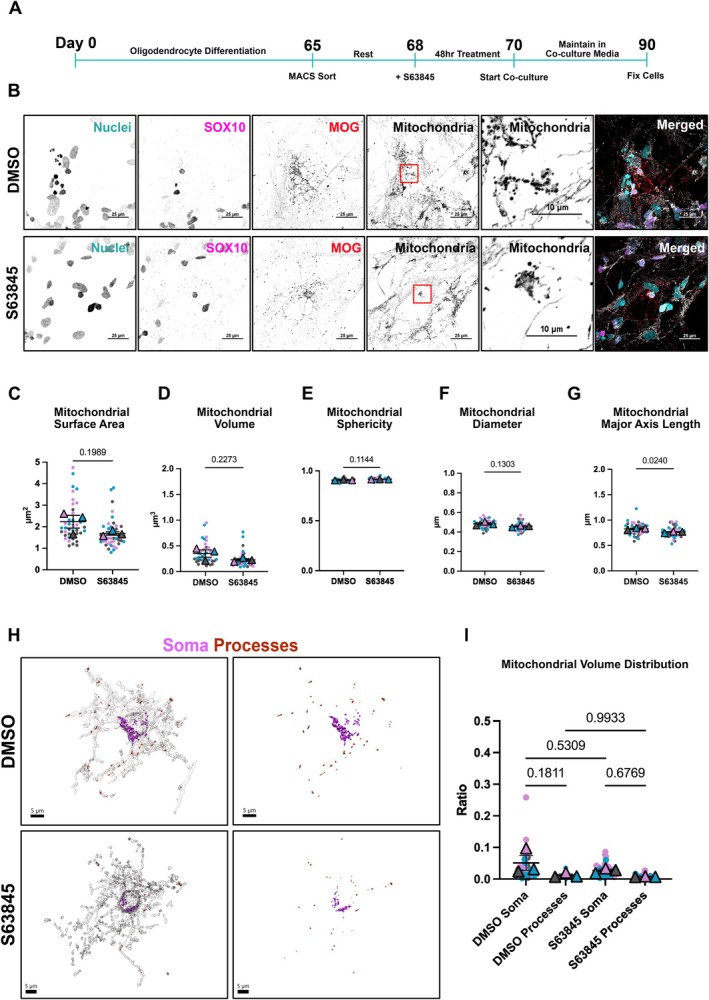
Pharmacological inhibition of MCL‐1 at early stages of development leads to mitochondrial morphology alterations in mature oligodendrocytes. (A) Timeline of oligodendrocyte differentiation, treatment with S63845, and co‐culture with motor neurons. At day 65 of differentiation, cells undergo cell sorting using the MACS cell separation system. Following a rest period, cells were treated with DMSO (vehicle) or S63845 for 48 h. At the 48 h timepoint, cells were cultured with motor neurons. Co‐culture was maintained until day 90. (B) Representative spinning disk confocal maximum intensity projections of immunofluorescent staining for nuclei (cyan), SOX10 (magenta), myelin oligodendrocyte glycoprotein (MOG) (red), and mitochondria (white) in mature oligodendrocytes that were previously treated with DMSO (vehicle) and S63845 (scale bar = 25 μm). Zoom in of mitochondria at the nucleus (scale bar = 10 μm). (C–G) Quantification of mitochondrial surface area, volume, sphericity, diameter, and major axis length. Each color represents a biological replicate (*n* = 3), each dot represents a cell (10–15 per *n*), each triangle represents mean of biological replicate, analyzed by student's *t*‐test, error bars represent mean ± SEM. (H) 3D reconstructions of mature oligodendrocytes and their mitochondrial network treated with DMSO (vehicle) and S63845. Mitochondria in magenta are in the cell's soma and mitochondria in red are in the cell's processes. (I) Distribution of mitochondria in mature oligodendrocytes. Ratio calculated by dividing the volume of the mitochondria in the soma (shown in magenta in G) or processes (shown in red in G) by the total volume of the mature oligodendrocyte. Each color represents a biological replicate (*n* = 3), each dot represents a cell (10–15 per *n*), each triangle represents mean of biological replicate, analyzed by one‐way ANOVA, error bars represent mean ± SEM. Conditions were blinded to the experimenter for 3D reconstructions.

**FIGURE 7:**
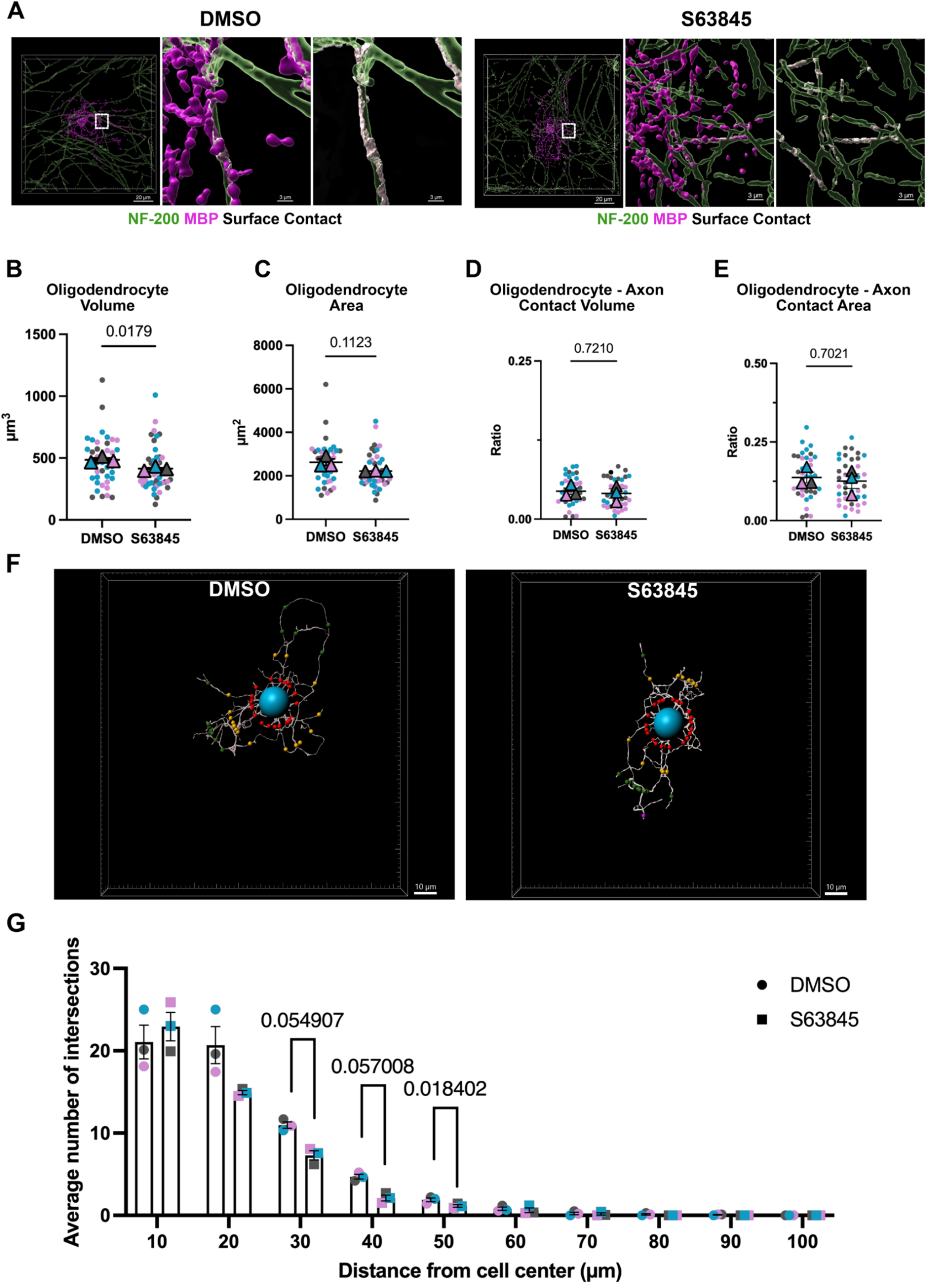
Using a human in vitro co‐culture system to investigate the long‐term effects of pharmacological inhibition of MCL‐1 in OPCs. (A) 3D reconstructions of myelin basic protein (MBP) positive cells (magenta), surrounding axons (green), and surface contact (white) in DMSO (vehicle) and S63845 treated cells. Scale bar = 20 μm. Scale bar = 3 μm in zoomed‐in panels. (B–C) MBP‐positive oligodendrocyte volume and area in DMSO (vehicle) and S63845 treated cells. Each color represents a biological replicate (*n* = 3), each dot represents a cell (10–15 per *n*), each triangle represents the mean of biological replicate, analyzed by student's *t*‐test, error bars represent mean ± SEM. Conditions were blinded to the experimenter for 3D reconstructions. (D) Ratio calculated by dividing the surface contact volume (white in Figure 5B) by the oligodendrocyte volume (magenta in Figure 5B). Each color represents a biological replicate (*n* = 3), each dot represents a cell (10–15 per *n*), each triangle represents the mean of the biological replicate, analyzed by student's *t*‐test, error bars represent mean ± SEM. Conditions were blinded to the experimenter for 3D reconstructions. (E) Ratio calculated by dividing the surface contact area (white in Figure 5B) by the oligodendrocyte area (magenta in Figure 5B). Each color represents a biological replicate (*n* = 3), each dot represents a cell (10–15 per *n*), each triangle represents the mean of the biological replicate, analyzed by student's *t*‐test, error bars represent mean ± SEM. Conditions were blinded to the experimenter for 3D reconstructions. (F) 3D filament reconstruction of MBP positive oligodendrocytes. Sholl analysis is indicated by different colored dots placed every 10 μm from cell center (blue dot) (scale bar = 10 μm). Conditions were blinded to the experimenter for 3D filament reconstructions. (G) Sholl analysis histogram demonstrating the average number of intersections (*y*‐axis) and distance from the cell's center (*x*‐axis). Each dot or square represents the mean number of intersections at a given distance (mean of 10–15 cells per biological replicate) in DMSO (vehicle) or S63845 treated cells, respectively. Each color represents a biological replicate (*n* = 3). Analyzed by student's *t*‐test, error bars represent mean ± SEM.

OPCs reorganize their mitochondrial network as they mature into myelinating oligodendrocytes in vivo (Bame and Hill 2024). In mice, myelinating oligodendrocytes have decreased mitochondrial volume compared to OPCs, and the mitochondria shift to a fragmented state (Bame and Hill 2024; Soares et al. 2024). This dramatic reorganization may be attributed to the shift in metabolic state that occurs with the growth of the cytoplasmic content and the formation of the myelin sheath. To examine the mitochondrial morphology in mature oligodendrocytes, we used a co‐culture system (Gil and Gama 2024). Immediately following a 48‐h treatment with S63845, OPCs were cultured with human iPSC‐derived motor neurons (Figure S7A,B). The co‐culture was maintained for 20 DIV and then fixed for imaging analysis (Figure 7A). To capture the entire cell, the co‐culture was stained with myelin oligodendrocyte glycoprotein (MOG), which is found on the cell surface of myelinating oligodendrocytes and external lamellae of myelin sheaths (Scolding et al. 1989). MOG, along with SOX10 staining, allowed for the identification of mature cells in the cell culture system and the generation of 3D reconstructions. As shown in the murine model, human oligodendrocytes have a fragmented mitochondrial network (Figure 6B) (Bame and Hill 2024; Soares et al. 2024). There are no significant changes to the mitochondrial surface area, volume, sphericity, and diameter (Figure 6C–F) in oligodendrocytes previously treated with S63845. Pharmacological inhibition of MCL‐1 at the OPC stages leads to decreased mitochondrial major axis length in mature oligodendrocytes (Figure 6G). To assess mitochondrial distribution in mature oligodendrocytes, we measured the volume of mitochondria within the soma or processes and divided it by the total volume of the oligodendrocyte to generate a ratio. Results indicate no significant alterations in mitochondrial distribution between the soma and the processes in vehicle‐treated cells (Figure 6H,I) and no significant differences in the volume of MOG‐positive cells (Figure S8).

### 2.8 Inhibition of MCL‐1 at Early Differentiation Stages Alters the Morphogenesis of Oligodendrocytes Without Disrupting Axon Contacts

A recent drug repurposing study found that miconazole, an antifungal agent, enhances the generation of human oligodendrocytes from human OPCs in vitro (Najm et al. 2015). To test if the oligodendrocyte differentiation system described here in combination with a neuron‐free nanofiber assay serves as a platform to test therapeutic agents, we treated cells cultured on nanofibers with miconazole for 20 days (Figure S9A). These findings suggest that miconazole may promote the growth of oligodendrocytes cultured in the nanofiber system, validating the potential use of this system to examine morphological changes during oligodendrocyte development (Figure S9B).

To understand if MCL‐1 and ACSL1 are required for the maintenance of mature oligodendrocyte processes, we compared the volume and area of oligodendrocytes following treatment with S63845 and Triacsin C (Figure S9A). We used miconazole as a positive control to compare morphological changes in oligodendrocytes as they matured on the nanofibers. Although not statistically significant, miconazole treatment showed an increased trend in oligodendrocyte volume and area; treatment with S63845 and Triacsin C did not lead to changes in the volume and area of oligodendrocytes (Figure S9C–E).

To examine if changes to gene expression of *MBP* detected in OPCs (Figure 5D) had an effect on the capacity of the OPCs to mature, we first treated OPCs with S63845 for 48 h, and continued to culture them in the motor neuron‐oligodendrocyte co‐culture system described previously for 20 days (Gil and Gama 2024). Axons from motor neurons were stained for neurofilament 200 (NF‐200), and oligodendrocytes were stained for MBP. 3D reconstructions were generated with Imaris, allowing for detailed visualization of oligodendrocytes and their processes. We used the Imaris XTension, Surface–Surface Contact Area, to visualize and quantify the contact between oligodendrocytes and axons (Figure 7A). Oligodendrocytes previously treated with S63845 exhibited lower cell volume compared to DMSO control (Figure 7B,C). We generated a ratio of the volume of the surface contact between the oligodendrocyte and surrounding axons divided by the volume of the oligodendrocyte (termed oligodendrocyte–axon contact). A similar ratio was generated to determine the area of the surface contact between the oligodendrocyte and surrounding axons divided by the area of the oligodendrocyte. The oligodendrocyte–axon contact volume and area are similar between the vehicle and S63845 treated groups (Figure 7D,E). Sholl analysis of MBP+ cells revealed that oligodendrocytes in the S63845 treated group have fewer intersections at 30, 40, and 50 μm distance from cell center, suggesting a loss in morphological complexity of these cells (Figure 7F,G).

## 3 Discussion

The developing brain has a high energetic demand that relies on glucose and lactate for normal function (Steiner 2020; Mergenthaler et al. 2013). Although understudied, it is known that the oxidation of fatty acids is also required (Ebert et al. 2003; Panov et al. 2014). Loss of lipid homeostasis has been described in Alzheimer's Disease pathology, illustrating the importance of this mechanism for human health (Yin 2023). The balance and interactions between glycolysis and FAO in the developing brain are poorly understood. FAO of short, medium, and long‐chain fatty acids occurs in the mitochondria (Bullón‐Vela et al. 2018; Houten and Wanders 2010). MCL‐1 is thought to modulate long‐chain fatty acid β‐oxidation by its interaction with ACSL1 (Wright et al. 2024). However, it is not known if this non‐apoptotic function of MCL‐1 is conserved in other cell types, such as oligodendrocytes, which are known to depend on MCL‐1 for proper development (Cleveland et al. 2021). Mature oligodendrocytes are mainly glycolytic, yet require FAO to uphold the metabolic support provided to axons (Fünfschilling et al. 2012; Ziabreva et al. 2010; Asadollahi et al. 2024). Here, we describe a platform to investigate human oligodendrocyte development. This platform allows for the isolation of human OPCs to examine their potential to mature into myelinating oligodendrocytes in neuron co‐culture or neuron‐free nanofiber assays. Outlining cell transition states in early development is critical for understanding key elements of differentiation and integration of oligodendrocytes into the neuronal system.

Proper integration of oligodendrocytes into the developing brain requires high energy production to support the expanding lipid‐rich membrane that composes the myelin sheath (Mergenthaler et al. 2013). Alterations to this process may lead to hyper‐ or hypomyelination, triggering severe white matter disorders (Adlkofer et al. 1995; Wolf et al. 2021). Regulation of mitochondrial dynamics and morphology is critical in supporting oligodendrocytes' cellular and energetic demands during development (Soares et al. 2024). Here, we present the first report of the characterization of mitochondrial morphology changes in an in vitro human system of oligodendrogenesis. Similar to findings in mouse tissue, mitochondria in OPCs have an elongated morphology and fragment as the cells mature (Bame and Hill 2024; Soares et al. 2024). This is accompanied by an apparent loss of mitochondrial volume in mature oligodendrocytes. These findings align with the concept that oligodendrocytes switch to glycolysis from OXPHOS, as glycolytic cells are thought to have fragmented mitochondria and cells that rely on OXPHOS have elongated mitochondria (Mishra and Chan 2016). The association between mitochondrial morphology and metabolic state is also dependent on the ultrastructure of the mitochondria, since mitochondria with an increased cristae volume and tight cristae structures have a higher capacity for efficient ATP production (Cogliati et al. 2016; Glancy et al. 2020; Mukherjee et al. 2021).

As OPCs develop and extend processes to explore the surrounding environment in search of axons to myelinate, mitochondrial distribution is expected to shift (Kirischuk et al. 1995). The absence of neurons in the OPC culture post‐sorting may prevent the growth of the processes, leading them to not have the same response as previously described in vivo (Bame and Hill 2024). Pharmacological inhibition of MCL‐1 at early stages of development did not alter mitochondrial morphology or distribution in OPCs in mature oligodendrocytes. However, ultrastructural alterations to the mitochondria, which are further indicative of the energetic state of the cell, require further examination (Jiang et al. 2017).

We observed a decrease in lipid droplet volume in etomoxir‐treated OPCs supplemented with palmitate in a glucose‐depleted environment. This was accompanied by a decrease in immunofluorescence intensity of OLIG2. Although some immunofluorescence intensity variability was observed in S63845 and Triacsin C‐treated groups, OLIG2 expression was not significantly altered under these treatment conditions. Loss of OLIG2 with etomoxir treatment at this stage of development may indicate a possible alteration to the function of OPCs. Inhibition of FAO by etomoxir can trigger an ER stress response, which has been shown to downregulate gene expression of *Olig2* (Gao et al. 2024). This crosstalk between FAO and ER stress response during membrane synthesis in oligodendrogenesis must be further investigated to understand myelin formation and oligodendrocyte differentiation.

We detected a loss in the complexity of MBP‐positive cells in response to MCL‐1 inhibition. Further investigation is needed to confirm possible dysfunction in myelin integrity as MBP is responsible for interacting with lipids to form tight adhesions of the cytosolic surfaces (Boggs 2006). The resolution provided by the spinning disk confocal images and 3D reconstructions presented here reveals that oligodendrocytes may still be in the early stages of development. Thus, the long‐term maintenance of the myelin sheath and axonal contact should be further investigated using ultrastructural imaging techniques. Additionally, it would be informative to determine if changes to FAO lead to alterations in established oligodendrocyte–axon contacts, as the energic requirements in myelinating oligodendrocytes differ from OPCs.

In our system and previously reported systems, it is technically challenging to isolate a pure population of human OPCs and maintain them for an extended period of time. This limitation has led to constraints on the experiments that can be performed to further investigate FAO, such as respirometry assays, metabolomics, and carbon tracing. Further understanding the interaction between MCL‐1 and ACSL1 in activating fatty acids in oligodendrocytes is needed. Currently, Triacsin C is the only commercially available inhibitor of ACSL1. Developing a specific inhibitor of the enzyme will further uncover the relationship between MCL‐1 and ACSL1. Live imaging modalities to visualize lipid droplet trafficking and accumulation following inhibition of MCL‐1, ACSL1, CPT1, and CPT2 should be used to better understand the reliance of OPCs on FAO for survival and function. Improved techniques are needed in the field of glia biology to further elucidate metabolic requirements that regulate the development of human oligodendrocytes, as they can best capture disease‐relevant features (Wang et al. 2023; Jhanji et al. 2024). Diseases like multiple sclerosis (MS), leukodystrophies, and other demyelinating disorders affect human oligodendrocytes specifically. While murine models are invaluable for understanding basic cellular processes, they often fail to fully capture the complexity of these diseases due to species differences in immune responses, neural development, and cell interactions. Isolating and maintaining human oligodendrocytes with myelination capacity in vitro facilitates the investigation of human‐specific pathophysiology, drug responses, and disease mechanisms.

The striking parallels in mitochondrial network reorganization between human and mouse models suggest that the core mechanisms governing mitochondrial morphology and function during the development of oligodendrocytes are evolutionarily conserved. Additionally, these results imply that specific mitochondrial events, such as the mitochondrial transport along oligodendrocyte projections vital for sustaining long‐distance signaling and cellular metabolism, appear to operate similarly in both species. Thus, integrating both models provides a more holistic approach to studying mitochondrial function and enables the identification of conserved mitochondrial mechanisms regulating oligodendrogenesis. The detection of species‐specific differences could also inform the development of more targeted and effective therapies for mitochondrial‐related disorders in humans.

## 4 Methods

### 4.1 Cell Line and Stem Cell Maintenance

H9 human embryonic stem cells passages number 40–45 (WiCell Research Institute, WA09, NIH Registration Number: 0062) were maintained in mTeSR1 (STEMCELL Technologies, 85850) on Matrigel‐coated (Corning, 354277) plates. Media was changed daily and passaged when they reached 60%–80% confluency.

### 4.2 Etoposide Treatment

H9 hESCs were treated with 20 μM etoposide (Sigma‐Aldrich, E1383) for 24 h. DMSO was used as the vehicle control.

### 4.3 Oligodendrocyte Differentiation

Differentiation was adapted from Douvaras and Fossati (2015). Detailed instructions for the differentiation can be found at Gil and Gama (2024). Basal media was used throughout the differentiation and was composed of DMEM/F12 (Thermo Fisher Scientific, 11320033), 1X Penicillin–Streptomycin (Thermo Fisher Scientific, 15‐140‐122), 1X MEM Non‐Essential Amino Acid (Thermo Fisher Scientific, 11‐140‐050), 1X GlutaMAX (Thermo Fisher Scientific), and 55 μM 2‐Mercaptoethanol (Bio‐Rad, 1610710).

H9 hESCs were dissociated using Accutase (Thermo Fisher Scientific, A1110501) and incubated at 37°C for 5 min. Accutase was diluted with DMEM/F12 medium. A cell lifter was used to remove cells from the well and fully dissociate by pipetting the mixture two to five times with a p1000 pipette. Cells were centrifuged at 200 ×*g* for 4 min at RT. Cells were plated at 8 × 10 (Knobloch et al. 2017) cells per well on a Matrigel‐coated 6‐well plate in mTeSR1 supplemented with 10 μM Y27632 (STEMCELL Technologies, 72307). The following day, mTeSR1 was replenished to remove Y27632. Medium changes were performed daily until 2 days following replating when cells reached 80% confluency. At this point, differentiation was started (Day 0) by feeding cells with neural induction medium (NIM) composed of basal media supplemented with 25 μg/mL insulin (Sigma‐Aldrich, I9278), 10 μM SB431542 (Reprocell, 04‐0010‐10), 250 nM LDN193189 (Reprocell, 04‐0074), and 100 nM Retinoic acid (Sigma‐Aldrich, R2625). Media were changed daily and SB431542, LDN193189, and Retinoic acid were added fresh each day. On Day 8, cells were fed with N2 medium. N2 medium was composed of basal medium with 1X N2 supplement (Thermo Fisher Scientific, 17502048), and 250 nM SAG Hedgehog pathway activator (STEMCELL Technologies, 73412). Medium was changed daily until Day 12. Retinoic acid and SAG were added fresh each day. On Day 12, aggregates were formed by mechanical dissociation. Old medium was removed from wells and replenished with 1 mL of N2B27 medium. N2B27 medium was composed of basal media supplemented with 1X N2 supplement, 1X B27 minus vitamin A supplement (Thermo Fisher Scientific, 12587010), 25 μg/mL insulin, 100 nM Retinoic acid, and 250 nM SAG. Aggregates were generated by using a cell lifter placed perpendicular to the bottom of the well and creating 20 parallel cuts into the cell layer to cover the entire well. The plate was turned 90° and 20 more cuts were made. Finally, the plate was turned 45° and an additional 20 cuts were made. The remaining cells were detached by scraping the well with the cell lifter. The cell clumps were broken up by pipetting with a P1000 three to five times. 500 μL of the cell aggregate suspension were transferred to one well of a 6‐well plate previously rinsed with Anti‐Adherence Rinsing Solution (STEMCELL Technologies, 07010) containing 2.5 mL of N2B27 media. Cell aggregates were fed every 2 days with N2B27 medium until Day 20. Aggregates were fed by transferring all the contents of a well into a 15 mL conical tube. Aggregates were allowed to sink to the bottom of the tube (approximately 3–5 min), and two‐thirds of the medium were aspirated. To prevent aggregates from clumping, they were gently pipetted three times with a P1000. Aggregates were added back to the well with fresh N2B27 medium. On Day 20, aggregates were fed with PDGF medium. PDGF medium was composed of basal media with 1X N2 supplement, 1X B27 minus vitamin A supplement, 10 ng/mL PDGF‐AA Protein (R&D Systems, 221‐AA), 10 ng/mL IGF‐I Protein (R&D Systems, 291‐G1‐200), 5 ng/mL HGF Protein (PeproTech, 315‐23), 10 ng/mL NT‐3 Protein (PeproTech, 450‐03), 60 ng/mL 3,3′,5‐Triiodo‐l‐thyronine (Sigma‐Aldrich, T28), 100 ng/mL Biotin (Sigma‐Aldrich, B4639), 1 μM cAMP (Sigma‐Aldrich, D0627), and 25 μg/mL insulin. On Day 30, round aggregates with a dark center were selected and seeded on a plate coated with poly‐l‐ornithine and laminin. Cells were fed every 2 days by gently removing the two‐thirds of the medium with a P1000 and replenishing with fresh medium using a P1000. Cells were fed until Day 65 with PDGF medium.

### 4.4 MACS to Isolate Oligodendrocyte Progenitor Cells

On Day 65 of oligodendrocyte differentiation, MACS was used to isolate OPCs. Cells and aggregates were dissociated by incubating with 2 mL Accutase at 37°C for 30 min. After the incubation, a p1000 was used to mechanically dissociate the cells by gently pipetting 7–10 times. Following mechanical dissociation, Accutase was diluted 1:1 with DMEM/F12 supplemented with 10 μM Y27632. Cell suspension was centrifuged at 200 ×*g* for 4 min at RT. Media was aspirated and cells were resuspended in DMEM/F12 supplemented with 100 μg/mL DNase (Worthington Biochemical, LS002006). After incubating in DNase, cell suspension was further dissociated by gently pipetting 7–10 times. Cells were passed through a 40 μm strainer. Flow‐through was collected and centrifuged at 200 ×*g* for 4 min at RT. Media was aspirated and cells were resuspended in 120 μL of cold 0.5% BSA made in 1X PBS. Cell suspension was incubated for 10 min in the refrigerator.

Forty microliter of the Anti‐A2B5 MicroBeads (Miltenyi Biotec, 130‐093‐392) were added to the cell suspension and incubated for 15 min in the refrigerator. Cells were washed by adding 1 mL of 0.5% BSA and spun down at 200 g for 4 min at RT. Supernatant was aspirated completely and resuspended in 500 μL of 0.5% BSA. Cell suspension was applied into MS Columns (Miltenyi Biotec, 130‐042‐201) attached to MiniMACS (Miltenyi Biotec, 130‐042‐102). Columns were washed with 1 mL of 0.5% BSA three times. The column was removed from the separator and placed on a suitable collection tube. To wash out cells from the column, 1 mL of 0.5% BSA was added and immediately flushed out the magnetically labeled cells by firmly and slowly pushing the plunger into the column to collect sorted OPCs. Sorted OPCs were seeded on a plate coated with poly‐l‐ornithine and laminin.

### 4.5 S63845 Treatment of Oligodendrocyte Progenitor Cells

Following sort, OPCs were treated with 2 μM S63845 (MedKoo, 406849) diluted in PDGF media for 48 h. DMSO (Sigma‐Aldrich, D2650) was used as a vehicle control. Media with S63845 was replenished at 24 h.

### 4.6 Glucose Depletion Experiment in Oligodendrocyte Progenitor Cells

Following sorting, cells were cultured in HBSS (Thermo Fisher Scientific, 14025092) supplemented with 100 μM BSA‐Palmitate Saturated Fatty Acid (Cayman Chemical, 29558) or BSA as the vehicle control (Cayman Chemical, 29556) for 24 h. During the 24‐h period, cells were treated with 50 μM etomoxir (Tocris, 4539), 2 μM S63845, or 2 μM Triacsin C (Tocris, 2472). DMSO was used as the vehicle control for Etomoxir, S63845, and Triacsin C. At the 24‐h timepoint, cells were treated with 4 μM BODIPY (Cayman Chemical, 25892) for 15 min to allow for lipid droplet detection. Cells were fixed with 4% paraformaldehyde following incubation with BODIPY.

### 4.7 Triacsin C Treatment of Oligodendrocyte Progenitor Cells

Following sorting, OPCs were treated with 2 μM Triacsin C diluted in PDGF media for 16 h. DMSO was used as a vehicle control. Cells were fixed with 4% paraformaldehyde at the 16‐h timepoint.

### 4.8 Motor Neuron Differentiation

Motor neuron differentiation protocol was adapted from Ziller et al. (2018). WTC11 human induced pluripotent stem cells (hiPSCs) were dissociated using Accutase and incubated at 37°C for 5 min. Accutase was diluted with DMEM/F12 medium. A cell lifter was used to remove cells from the well and fully dissociate by pipetting the mixture two to five times with a p1000 pipette. Cells were centrifuged at 200 ×*g* for 4 min at RT. Cells were plated at 8 × 10 (Knobloch et al. 2017) cells per well on a Matrigel‐coated 6‐well plate in E8 supplemented with 10 μM Y27632. The following day, E8 was replenished to remove Y27632. Medium changes were performed daily until 1 day following replating when cells reached 80%–90% confluency. At this point, differentiation was started (Day 0) by feeding cells with motor neuron induction medium (MNIM) composed of 50% DMEM/F12, 50% Neurobasal media (Thermo Fisher Scientific, 21103049), 1X Penicillin‐Streptomycin, 1X MEM Non‐Essential Amino Acid, 1X GlutaMAX, 1X N2 supplement, and 1X B27 minus vitamin A supplement. MNIM was supplemented with 10 μM SB431542, 100 nM LDN193189, 1 μM SAG, and 1 μM Retinoic acid. Media were changed daily, and SB431542, LDN193189, SAG, and Retinoic acid were added fresh each day. On Day 6, cells were transitioned to MNIM supplemented with 4 μM SU5402 (DNSK International, 215542‐92‐3), 5 μM DAPT (DNSK International, 208255‐80‐5), 1 μM SAG, and 1 μM Retinoic acid. Media were changed daily until Day 13, and SU5402, DAPT, SAG, and Retinoic acid were added fresh each day. On Day 14, neurons were dissociated using Accutase and incubated at 37°C for 10 min. Accutase was diluted with DMEM/F12 medium. A cell lifter was used to remove cells from the well and fully dissociate by pipetting the mixture two to five times with a p1000 pipette. Cells were centrifuged at 200 ×*g* for 4 min at RT. Cells were plated at 1 × 10 (Brinkmann et al. 2025) cells per well on a poly‐l‐ornithine/laminin‐coated 6‐well plate in motor neuron maturation media (MNMM) supplemented with 10 μM Y27632. MNMM was composed of Neurobasal media, 1X Penicillin‐Streptomycin, 1X MEM Non‐Essential Amino Acid, 1X GlutaMAX, 1X N2 supplement, 1X B27 minus vitamin A supplement, 0.2 μg/mL Ascorbic Acid (Sigma‐Aldrich, A4403), 10 ng/mL CNTF (R&D Systems, 257‐NT‐010), 10 ng/mL BDNF (Peprotech, 11166‐BD‐010), and 10 ng/mL GDNF (R&D Systems, 11166‐BD‐010). The following day, MNMM was replenished to remove Y27632. Cells were fed every 2 days until Day 22. To prepare neurons for co‐culture, cells were replated at 1 × 10 per mL (Asadollahi et al. 2024) on poly‐l‐ornithine/laminin‐coated plates in MNMM supplemented with 10 μM Y27632 on Day 23. The following day, MNMM was replenished to remove Y27632. On Day 26, motor neurons were transitioned to 50% MNMM and 50% co‐culture media. Co‐culture media was composed of Neurobasal media, 1X Penicillin‐Streptomycin, 1X MEM Non‐Essential Amino Acid, 1X GlutaMAX, 1X N2 supplement, 1X B27 minus vitamin A supplement, 20 μg/mL Ascorbic Acid, 10 ng/mL CNTF, 10 ng/mL BDNF, 10 ng/mL GDNF, 60 ng/mL T3, 100 ng/mL biotin, 1 μM cAMP, and 25 μg/mL Insulin.

### 4.9 Co‐Culture Maintenance

Following S63845 treatment, OPCs were replated at 1 × 10 per mL (Asadollahi et al. 2024) onto motor neurons. Co‐culture was maintained in co‐culture media by feeding cells every other day until Day 20.

### 4.10 Neuron‐Free Nanofiber Assay

Nanofiber inserts (Sigma‐Aldrich, Z694614‐12EA) were removed from their packaging in a sterile environment and placed into the wells of a 12‐well plate with the fibers oriented upright. Each well was coated with 1 mL of 50 μg/mL poly‐l‐ornithine in dH2O and incubated at 37°C overnight. Poly‐l‐ornithine–coated plates were stored at 37°C for up to 4 days. Following incubation, the coating solution was aspirated, and the plate was air‐dried for 5 min before adding 1 mL of 20 μg/mL natural mouse laminin in DMEM/F12 per well. The plate was incubated at 37°C for at least 4 h, after which poly‐l‐ornithine/laminin‐coated plates could be stored at 37°C for up to 2 days.

For nanofiber culture, OPCs were counted following sorting, and a suspension was prepared at a density of 1 × 10^4^ cells per well in 200 μL Glia media supplemented with 10 μM Y27632. Glia media was composed of DMEM/F12, 1X Penicillin‐Streptomycin, 1X MEM Non‐Essential Amino Acid, 1X GlutaMAX, 1X N2 supplement, 1X B27 minus vitamin A supplement, 100 ng/mL Biotin, 1 μM cAMP, 25 μg/mL Insulin, 10 mM HEPES (Thermo Fisher Scientific, 15630080), and 20 μg/mL Ascorbic Acid. Laminin solution was aspirated from the nanofibers, and the OPC suspension was carefully pipetted onto the top surface of the nanofiber inserts, ensuring that the suspension did not migrate beneath the insert. Plates were incubated at 37°C for 2 h to allow cells to settle. Subsequently, 800 μL of Glia media containing 10 μM Y27632 was gently added to each well, resulting in a final volume of 1 mL. The following day, media were replaced with fresh Glia media to remove Y27632. Cultures were maintained by replenishing Glia media every 2 days until Day 30.

### 4.11 Miconazole Treatment of Oligodendrocytes in Neuron‐Free Nanofiber Assay

At Day 10 of nanofiber culture, media was removed and replaced with Glia media supplemented with 1 μM miconazole (Sigma‐Aldrich, PHR1618‐1G), with DMSO used as a vehicle control. Cultures were subsequently fed every other day with Glia media containing 1 μM miconazole until Day 30. On Day 30, cells were fixed with 4% paraformaldehyde.

### 4.12 S63845 Treatment of Oligodendrocyte Progenitor Cells Neuron‐Free Nanofiber Assay

At Day 28 of nanofiber culture, media was removed and replaced with Glia media supplemented with 2 μM S63845, with DMSO used as a vehicle control. Glia media with S63845 was replenished at 24 h. Following 48 h of treatment, cells were fixed with 4% paraformaldehyde.

### 4.13 Triacsin C Treatment of Oligodendrocytes in Neuron‐Free Nanofiber Assay

At Day 29 of nanofiber culture, media was removed and replaced with Glia media supplemented with 2 μM Triacsin C, with DMSO used as a vehicle control. Following 16 h of treatment, cells were fixed with 4% paraformaldehyde.

### 4.14 Western Blot

Cells were harvested in lysis buffer composed of PhosSTOP (Roche, 04906837001), PIC (Roche, 04693116001), and PMSF (RPI, P20270‐10.0) diluted in 1% Triton (Sigma‐Aldrich, T9284). Cells were sonicated to ensure complete cell lysis. 15 μg of protein was loaded and run in 4%–20% Mini‐Protean TGX precast protein gels (Bio‐Rad, 4561094) in Tris‐Gly‐SDS buffer. Gels were transferred onto polyvinylidene difluoride membranes (Bio‐Rad, 1620177) at 4°C overnight. The membrane was blocked in 5% milk (RPI, M17200500) diluted in TBST for 1 h at room temperature on a benchtop rocker. Primary antibodies were incubated at 4°C overnight on a benchtop rocker. HRP‐conjugated secondary antibodies against mouse or rabbit IgG were incubated for 1 h at room temperature on a benchtop rocker. Blots were developed with ECL Plus Reagent (Thermo Fisher Scientific, 32106) and imaged on a Chemiluminescent Imager. Bands were quantified with Image Studio Lite.

### 4.15 RNA Extraction and cDNA Synthesis

Cells were harvested in TRIzol Reagent (Invitrogen, 15596018). RNA isolation was performed following the Invitrogen TRIzol Reagent Protocol. RNA was incubated with DNase I (New England Biolabs, B0303S) at 37°C for 10 min. The reaction was inactivated by adding 0.1 M EDTA (EMD Millipore, 324506) and incubated for 10 min at 75°C. Ten microliter of this volume was used with the High‐Capacity cDNA Reverse Transcription Kit (Thermo‐Fisher Scientific, 4368814) to obtain cDNA.

### 4.16 Quantitative RT PCR

One microgram of cDNA sample was used to run RT‐qPCR on the QuantStudio 3 Real‐Time PCR machine with SYBR Green master mix (Thermo Fisher Scientific, 4309155). Manufacturer's instructions were used to set up the assay.

### 4.17 Immunofluorescence

Cells were fixed with 4% paraformaldehyde (Electron Microscopy Sciences, 15710‐S) in 1X PBS for 30 min at room temperature. Cells were incubated in blocking and permeabilization solution composed of 0.3% Triton (Sigma‐Aldrich, T9284) and 5% normal donkey serum (Jackson ImmunoResearch, 017‐000‐121) in 1X TBS for 1 h at room temperature. Primary antibodies were diluted in blocking and permeabilization solution and incubated at 4°C overnight. Cells were washed three times with 1X TBS followed by incubation in secondary antibody diluted in blocking and permeabilization solution for 1 h at room temperature. Cells were washed three times with 1X TBS. Cells were incubated with Hoechst (1 μg/mL) (Thermo Fisher Scientific, H3570) for 10 min. Cells were washed once with 1X TBS and sealed with Fluoromount‐G slide mounting medium (Thermo Fisher Scientific, 00‐4958‐02). Cells were imaged on the Nikon W1 Spinning Disk Confocal 100X Plan Apo 1.49 NA Oil objective and a Prime 95B CMOS camera. Cells were imaged on the Nicon W1 super‐resolution spinning‐disk confocal microscope using Optical Photon Reassignment (SoRa). 100X Plan Apo 1.49 NA Oil objective and a Prime 95B CMOS camera were used. Quantification of microscopy images was performed in NIS‐Elements (Nikon) or Imaris (Oxford Instruments). Surface–Surface Contact Area and Filament Sholl Analysis Imaris XTensions were used for image analyses.

### 4.18 Statistical Analysis

All experiments were performed with a minimum of three biological replicates. Statistical significance was determined by student's *t*‐test, one‐way ANOVA, or two‐way ANOVA as appropriate for each experiment. A detailed statistical report can be found in Table S1. GraphPad Prism v10 was used for all statistical analyses (SAS Institute: Cary, NC, USA) and data visualization. Error bars in all graphs represent standard error of the mean unless otherwise noted in the figure legend. No outliers were removed from the analyses. For all statistical analyses, a significant difference was accepted when *p* < 0.05.

**Table:** Antibodies.

Antibody	Species	Dilution	Company	Catalog number
OLIG2	Rabbit	IF—1:500	Millipore	AB9610
OLIG2	Mouse	IF—1:500 WB—1:1000	Millipore	MABN50
PDGFRα	Goat	IF—1:100	R&D Systems	AF‐307‐NA
MBP	Rat	IF—1:100	Millipore	MAB386
Mitochondria	Rabbit	IF—1:200	Abcam	ab92824
ISL1/2	Mouse	IF—1:200	DSHB	39.4D5
NF‐200	Mouse	IF—1:200	Millipore	N5389
Beta‐III‐Tubulin	Rabbit	IF—1:200	Abcam	ab18207
MOG	Goat	IF—1:500	R&D Systems	AF2395
SOX10	Rabbit	IF—1:200 WB—1:1000	Abcam	ab155279
MCL‐1	Rabbit	WB—1:1000	Cell Signaling Technologies	94296S
α‐Tubulin	Mouse	WB—1:1000	Sigma‐Aldrich	T9026
Cleaved Caspase‐3	Rabbit	IF—1:200	Cell Signaling Technologies	9661
GFAP	Rabbit	IF—1:200	Dako	GA52461‐2
Donkey anti‐Goat IgG (H+L) Highly Cross‐Adsorbed Secondary Antibody, Alexa Fluor Plus 647	Donkey	IF—1:500	Invitrogen	A32849
Donkey anti‐Rat IgG (H+L) Highly Cross‐Adsorbed Secondary Antibody, Alexa Fluor 647	Donkey	IF—1:500	Invitrogen	A78947
Donkey anti‐Rabbit, Alexa Fluor Plus 405, Secondary Antibody	Donkey	IF—1:500	Invitrogen	PIA48258
Donkey anti‐Mouse IgG (H+L) Highly Cross‐Adsorbed Secondary Antibody, Alexa Fluor 488	Donkey	IF—1:500	Invitrogen	A‐21202
Donkey anti‐Mouse IgG (H+L) Highly Cross‐Adsorbed Secondary Antibody, Alexa Fluor 568	Donkey	IF—1:500	Invitrogen	A10037
Donkey anti‐Rabbit IgG (H+L) Highly Cross‐Adsorbed Secondary Antibody, Alexa Fluor 488	Donkey	IF—1:500	Invitrogen	A‐21206
Peroxidase AffiniPure Donkey Anti‐Mouse IgG (H+L)	Donkey	WB—1:5000	Jackson ImmunoResearch	715‐035‐150
Peroxidase AffiniPure Donkey Anti‐Rabbit IgG (H+L)	Donkey	WB—1:5000	Jackson ImmunoResearch	711‐035‐152

**Table:** qPCR Primers.

Primer	Sequence (5′‐3′)
GPI Forward	GTGTACCTTCTAGTCCCGCC
GPI Reverse	GGTCAAGCTGAAGTGGTTGAAGC
GADPH Forward	ACAACTTTGGTATCGTGGAAGG
GADPH Reverse	GCCATCACGCCACAGTTTC
MCL1 Forward	TGCTTCGGAAACTGGACATCA
MCL1 Reverse	TAGCCACAAAGGCACCAAAAG
GFAP Forward	GGCAAAAGCACCAAAGACGG
GFAP Reverse	GGCGGCGTTCCATTTACAAT
MBP Forward	AGCGCACCTGTGATTGATAG
MBP Reverse	AAGACGCGTTTTGGCATCAC
ACSL1 Forward	CCATGAGCTGTTCCGGTATTT
ACSL1 Reverse	CCGAAGCCCATAAGCGTGTT
CPT2 Forward	CTGGAGCCAGAAGTGTTCCAC
CPT2 Reverse	AGGCACAAAGCGTATGAGTCT
CPT1A Forward	GCGCCCCTTGTTGGATGAT
CPT1A Reverse	CCACCATGACTTGAGCACCAG
OLIG2 Forward	AAGGCAGTTGCTGTGGAAACG2
OLIG2 Reverse	GCAAACAGCTTAGCATTGCGG3
PDGFRA Forward	TGGCAGTACCCCATGTCTGAA
PDGFRA Reverse	CCAAGACCGTCACAAAAAGGC
SOX10 Forward	CCTCACAGATCGCCTACACC
SOX10 Reverse	CATATAGGAGAAGGCCGAGTAGA

## Author Contributions

M. G. and V. G. conceived the study, designed experiments, interpreted data, and wrote the manuscript. V. G. supervised and funded the research. M. G. designed and carried out all the cell biology experiments with technical assistance from M. R. H. All authors edited the document.

## Funding

This work was supported by the National Institutes of Health (2R35GM128915‐06) and the Howard Hughes Medical Institute (GT15720).

## Conflicts of Interest

The authors declare no conflicts of interest.

## Supporting information


**Figure S1:** Validation of oligodendrocyte lineage cell population after cell sort. (A) Spinning disk confocal maximum intensity projections of immunofluorescent staining for nuclei (cyan) and OLIG2 (magenta) in cells after sorting (scale bar = 100 μm). (B) Chart outlining the percent of OLIG2 positive cells over nuclei in each biological replicate following cell sort. (C) Spinning disk confocal maximum intensity projections of immunofluorescent staining for nuclei (white) and GFAP (red) in cells after sorting (scale bar = 100 μm).

**Figure S2:** Representative images of cleaved caspase 3‐positive neural progenitor cells. (A) Spinning disk confocal maximum intensity projections of immunofluorescent staining for nuclei (cyan) and cleaved caspase 3 (green) in neural progenitor cells after etoposide treatment (scale bar = 25 μm).

**Figure S3:** Volume of PDGFRα positive cells. (A) Volume of platelet‐derived growth factor receptor alpha (PDGFRα) positive cells treated with DMSO (vehicle) and S63845. Each color represents a biological replicate (*n* = 3), each dot represents a cell (10–15 per *n*), each triangle represents mean of biological replicate, analyzed by student's *t*‐test, error bars represent mean ± SEM. Conditions were blinded to experimenter for 3D reconstructions.

**Figure S4:** Quantification of OLIG2 and PDGFRα immunofluorescence intensity following treatment with etomoxir, S63845, and Triacsin C in glucose depleted state. (A–F) *Z*‐score quantification of average immunofluorescence intensity of OLIG2 and PDGFRα. Each color represents a biological replicate (*n* = 3), each square or triangle represents the mean of the biological replicate, analyzed using an ordinary two‐way ANOVA, error bars represent mean ± SEM.

**Figure S5:** Pharmacological inhibition of ACSL leads to robust alterations to mitochondrial morphology and localization. (A) Representative spinning disk confocal maximum intensity projections of immunofluorescent staining for nuclei (cyan), oligodendrocyte transcription factor 2 (OLIG2) (green), platelet‐derived growth factor receptor alpha (PDGFRα) (magenta), and mitochondria (red) in DMSO (vehicle) and Triacsin C treated OPCs (scale bar = 25 μm zoom in of mitochondria at the nucleus [scale bar = 10 μm]). (B) Quantification of the distance between mitochondria and nucleus. Each color represents a biological replicate (*n* = 3), each dot represents a cell (10–15 per *n*), each triangle represents mean of biological replicate, analyzed by student's *t*‐test, error bars represent mean ± SEM.

**Figure S6:** Protein level of OLIG2 and SOX10 in OPCs are not altered following inhibition of MCL‐1. (A) Western blot of OLIG2 levels in OPCs following DMSO (vehicle) and S63845 treatment (left panel) and band density quantification (right panel). Each dot on graphs represents a biological replicate (*n* = 3), analyzed by students *t*‐test, error bars represent mean ± SEM. (B) Western blot of SOX10 levels in OPCs following DMSO (vehicle) and S63845 treatment (left panel) and band density quantification (right panel). Each dot on graphs represents a biological replicate (*n* = 3), analyzed by student's *t*‐test, error bars represent mean ± SEM.

**Figure S7:** Validation of motor neuron differentiation and representative images of co‐culture. (A) Representative spinning disk confocal maximum intensity projections of immunofluorescent staining for nuclei (cyan), ISL1/2 (magenta), and βIII‐tubulin (green) in motor neurons derived from human induced pluripotent stem cells (scale bar = 25 μm). (B) Representative spinning disk confocal maximum intensity projections of immunofluorescent staining for nuclei (blue), Neurofilament‐200 (NF‐200) (green), and myelin basic protein (MBP) (magenta) in Day 90 co‐cultures with cells that were previously treated as OPCs with DMSO (vehicle) or S63845 (scale bar = 25 μm).

**Figure S8:** Volume of MOG‐positive cells. Volume of myelin oligodendrocyte glycoprotein (MOG) positive cells in Day 90 co‐cultures with cells that were previously treated as OPCs with DMSO (vehicle) and S63845. Each color represents a biological replicate (*n* = 3), each dot represents a cell (10–15 per *n*), each triangle represents mean of biological replicate, analyzed by student's *t*‐test, error bars represent mean ± SEM. Conditions were blinded to experimenter for 3D reconstructions.

**Figure S9:** Using a neuron‐free nanofiber assay to evaluate pharmacological inhibition of MCL‐1 and ACSL1. (A) Timeline of oligodendrocyte differentiation, seeding on nanofibers, treatment with miconazole, S63845, and Triascin C. At Day 65 of differentiation, cells undergo cell sorting using the MACS cell separation system. Cells were directly seeded onto nanofibers after MACS sorting, commencing nanofiber culture Day 0. At Day 10, a first set of cells were treated with miconazole or DMSO (vehicle) and treatment continued for 20 days until collection. A second set of cells were treated with S63845 or DMSO (vehicle) for 48 h at Day 28. At Day 29, third set of cells were treated with Triacsin C or DMSO (vehicle) for 16 h. Cells were fixed at Day 30 following treatments. (B) Representative SoRa maximum intensity projections of immunofluorescent staining for nanofibers (white) and MBP (magenta) in oligodendrocytes treated with DMSO (vehicle) and miconazole (scale bar = 25 μm). Zoom‐in of oligodendrocyte process on nanofiber (scale bar = 10 μm). (C) 3D reconstructions of MBP‐positive oligodendrocytes cultured on nanofibers with miconazole, S63845, Triacsin C, and their respective DMSO vehicle controls (scale bar = 15 μm). (D) Quantification of MBP‐positive oligodendrocyte volume in miconazole, S63845, Triacsin C, and respective DMSO (vehicle) treated cells. Each color represents a biological replicate (*n* = 3), each dot represents a cell (6–11 per *n*), each triangle represents the mean of biological replicate analyzed by ordinary one‐way ANOVA, error bars represent mean ± SEM. Conditions were blinded to the experimenter for 3D reconstructions. (E) Quantification of MBP‐positive oligodendrocyte area in Miconazole, S63845, Triacsin C, and respective DMSO (vehicle) treated cells. Each color represents a biological replicate (*n* = 3), each dot represents a cell (6–11 per *n*), each triangle represents the mean of biological replicate analyzed by ordinary one‐way ANOVA, error bars represent mean ± SEM. Conditions were blinded to the experimenter for 3D reconstructions.

**Table S1:** Detailed statistical report.

## Data Availability

The data that support the findings of this study are available on request from the corresponding author. The data are not publicly available due to privacy or ethical restrictions.
