# Supplementary figures and images for: A Human Model of Oligodendrocyte Development Shows MCL‐1 Influences Oligodendrocyte Morphogenesis
Source: Glia. 2025 Dec 19;74(2):e70128. doi: 10.1002/glia.70128 (PMC12717335; doi:10.1002/glia.70128)

Supplementary Figure 1

A

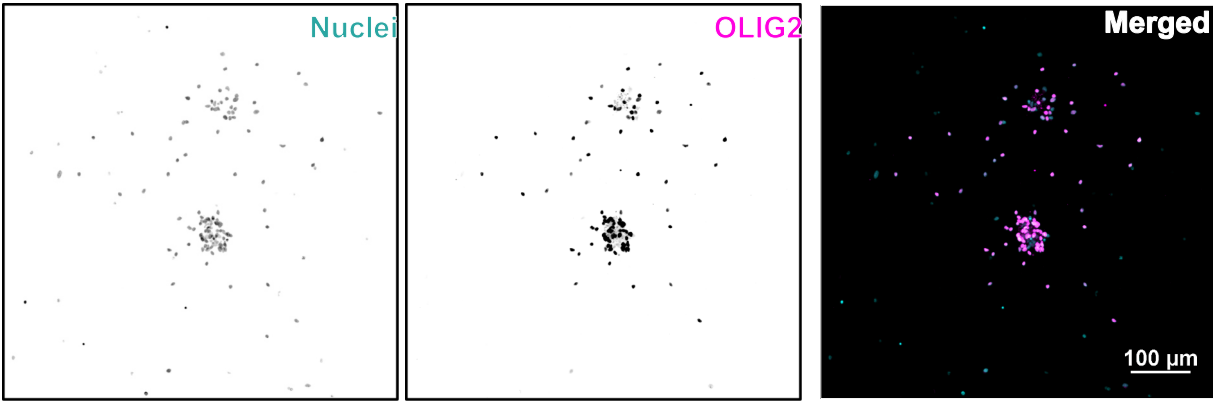

B

| Biological Replicate # | % of OLIG2/Nuclei |
|------------------------|-------------------|
| 1                      | 71%               |
| 2                      | 57%               |
| 3                      | 80%               |
| 4                      | 80%               |
| Average                | 72%               |

C

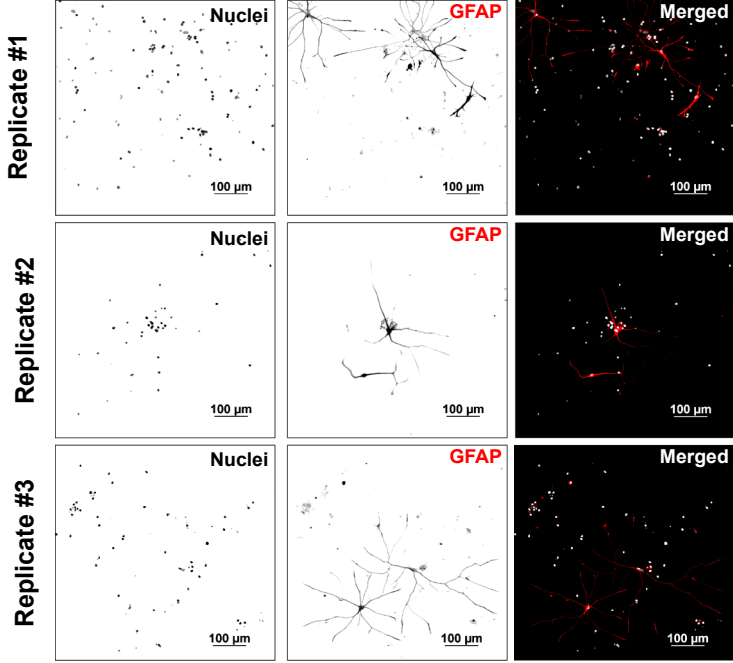

Supplement: Supplementary file 1 — Figure S1: Validation of oligodendrocyte lineage cell population after cell sort. (A) Spinning disk confocal maximum intensity projections of immunofluorescent staining for nuclei (cyan) and OLIG2 (magenta) in cells after sorting (scale bar = 100 μm). (B) Chart outlining the percent of OLIG2 positive cells over nuclei in each biological replicate following cell sort. (C) Spinning disk confocal maximum intensity projections of immunofluorescent staining for nuclei (white) and GFAP (red) in cells after sorting (scale bar = 100 μm). [file GLIA-74-0-s002.pdf]

**A**

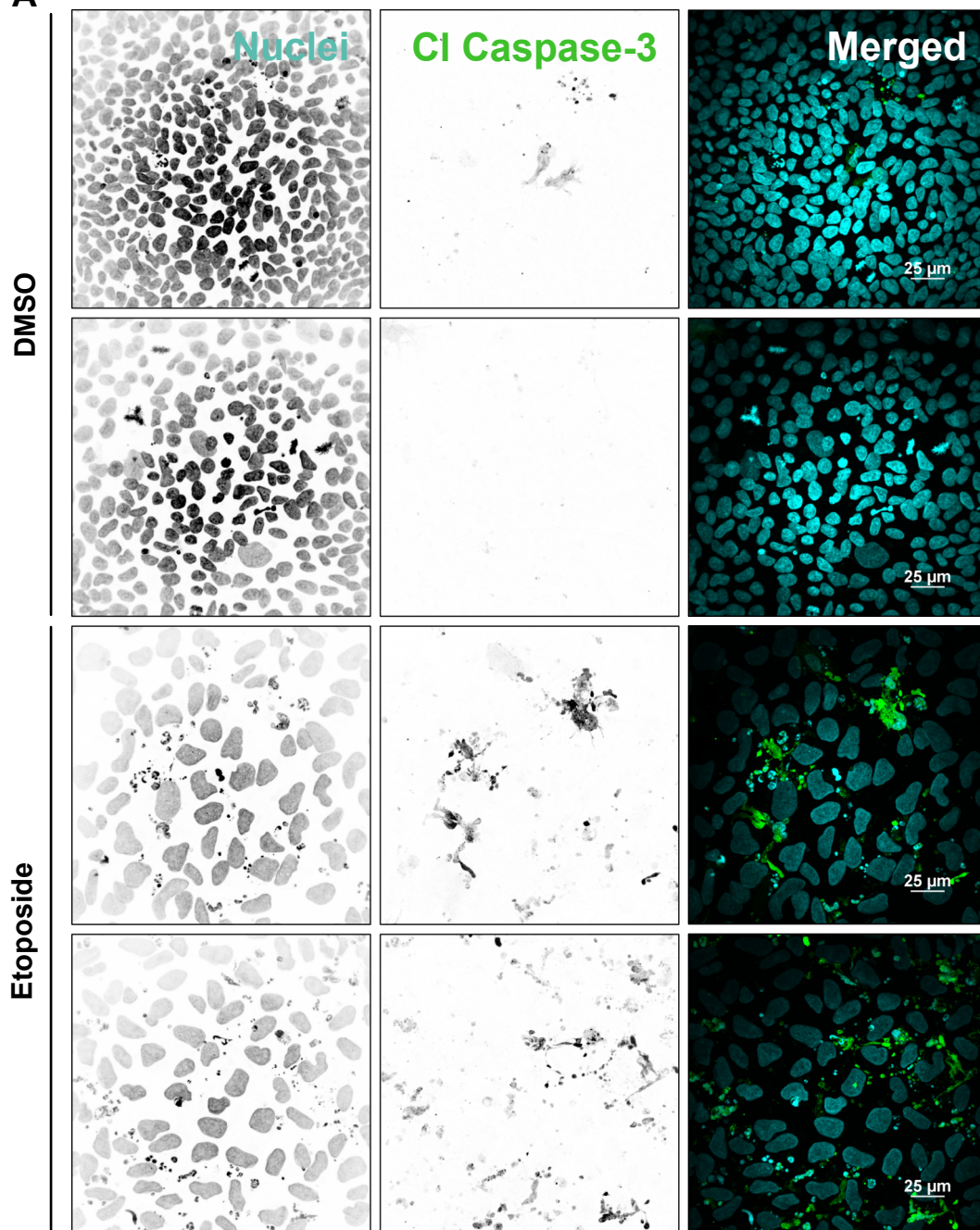

Supplement: Supplementary file 2 — Figure S2: Representative images of cleaved caspase 3‐positive neural progenitor cells. (A) Spinning disk confocal maximum intensity projections of immunofluorescent staining for nuclei (cyan) and cleaved caspase 3 (green) in neural progenitor cells after etoposide treatment (scale bar = 25 μm). [file GLIA-74-0-s001.pdf]

Supplementary Figure 3

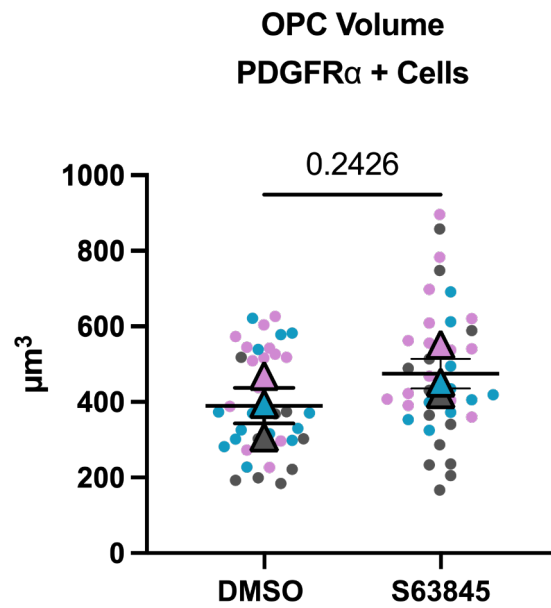

Supplement: Supplementary file 3 — Figure S3: Volume of PDGFRα positive cells. (A) Volume of platelet‐derived growth factor receptor alpha (PDGFRα) positive cells treated with DMSO (vehicle) and S63845. Each color represents a biological replicate (n = 3), each dot represents a cell (10–15 per n), each triangle represents mean of biological replicate, analyzed by student's t‐test, error bars represent mean ± SEM. Conditions were blinded to experimenter for 3D reconstructions. [file GLIA-74-0-s008.pdf]

Supplementary Figure 4

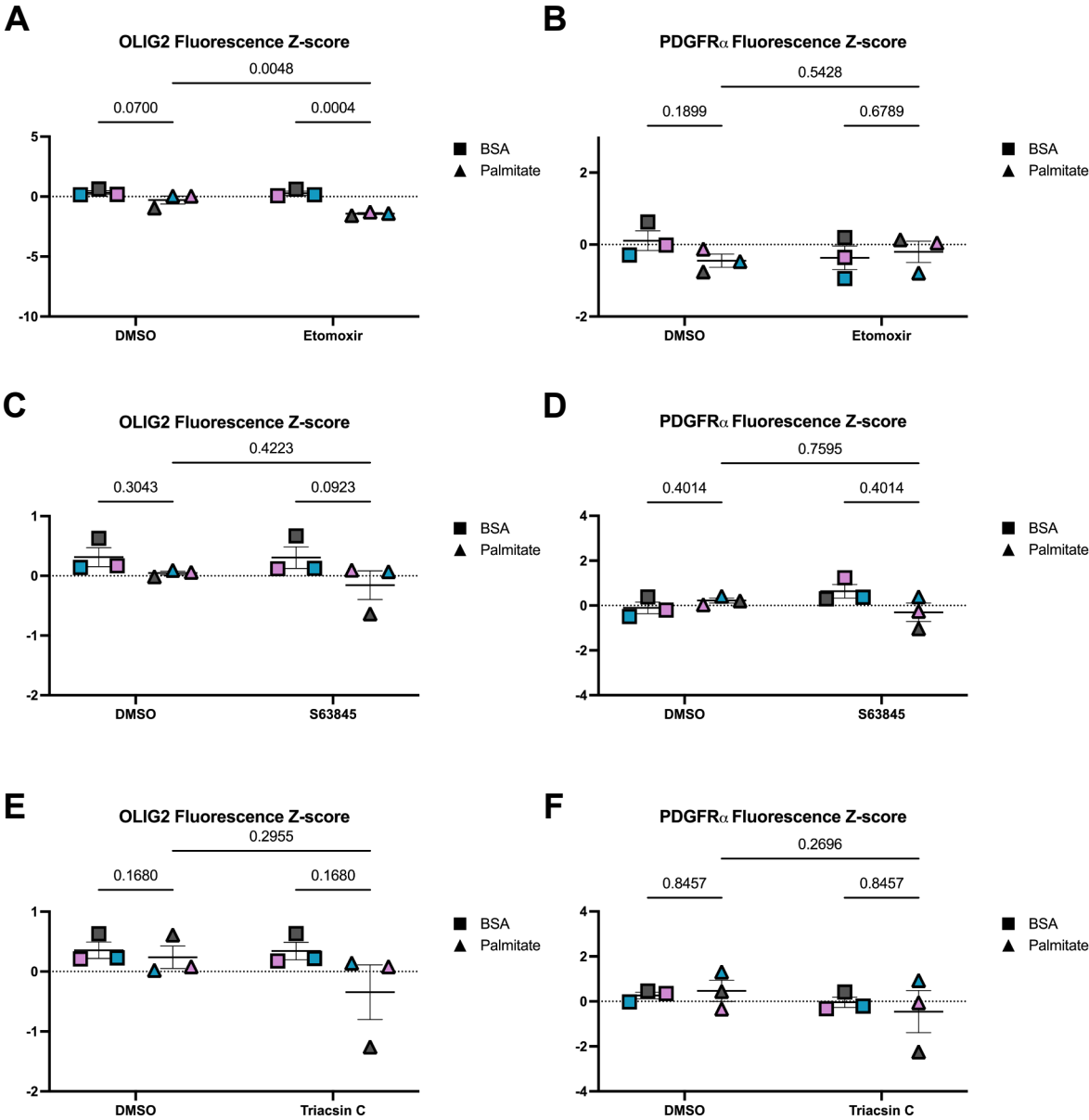

Supplement: Supplementary file 4 — Figure S4: Quantification of OLIG2 and PDGFRα immunofluorescence intensity following treatment with etomoxir, S63845, and Triacsin C in glucose depleted state. (A–F) Z‐score quantification of average immunofluorescence intensity of OLIG2 and PDGFRα. Each color represents a biological replicate (n = 3), each square or triangle represents the mean of the biological replicate, analyzed using an ordinary two‐way ANOVA, error bars represent mean ± SEM. [file GLIA-74-0-s010.pdf]

Supplementary Figure 6

A

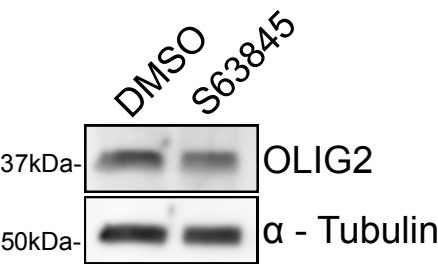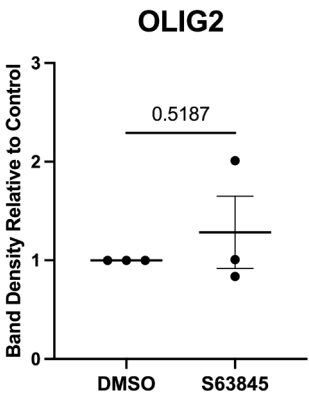

B

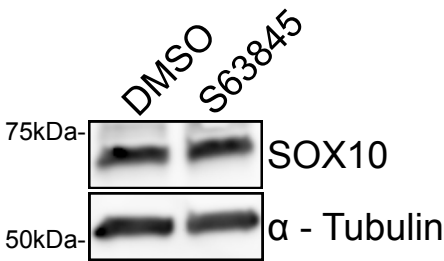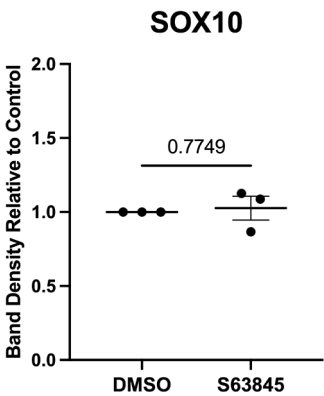

Supplement: Supplementary file 6 — Figure S6: Protein level of OLIG2 and SOX10 in OPCs are not altered following inhibition of MCL‐1. (A) Western blot of OLIG2 levels in OPCs following DMSO (vehicle) and S63845 treatment (left panel) and band density quantification (right panel). Each dot on graphs represents a biological replicate (n = 3), analyzed by students t‐test, error bars represent mean ± SEM. (B) Western blot of SOX10 levels in OPCs following DMSO (vehicle) and S63845 treatment (left panel) and band density quantification (right panel). Each dot on graphs represents a biological replicate (n = 3), analyzed by student's t‐test, error bars represent mean ± SEM. [file GLIA-74-0-s006.pdf]

Supplementary Figure 7

A

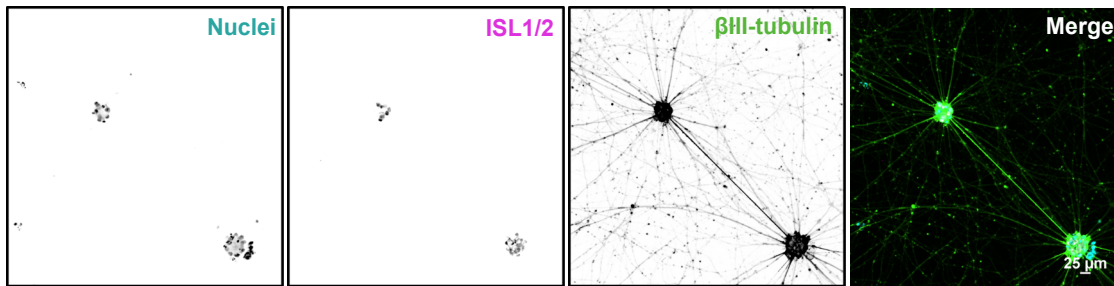

B

DMSO  
Representative Images

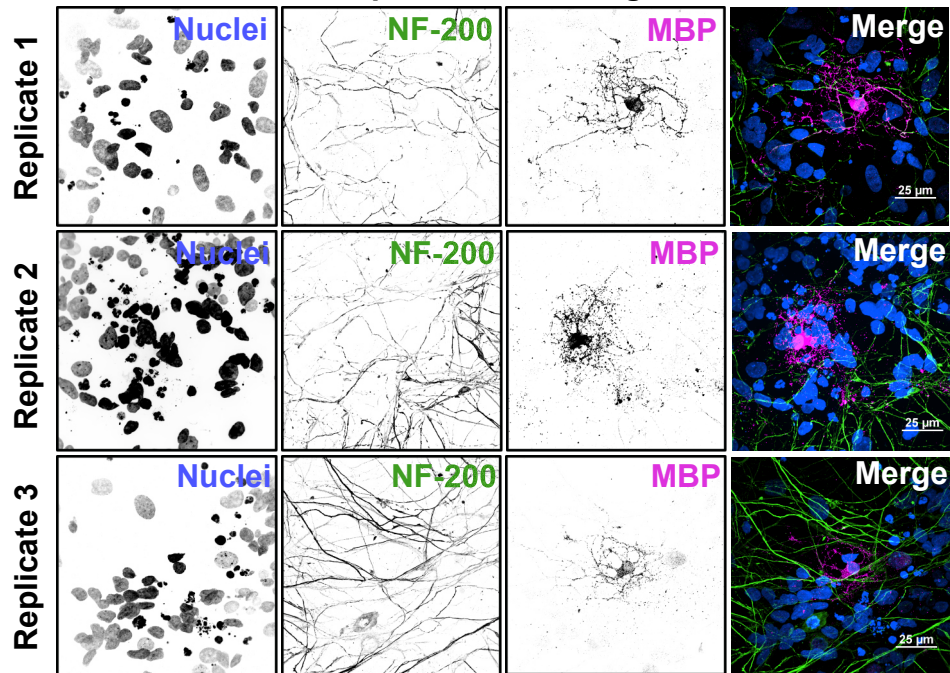

S63845  
Representative Images

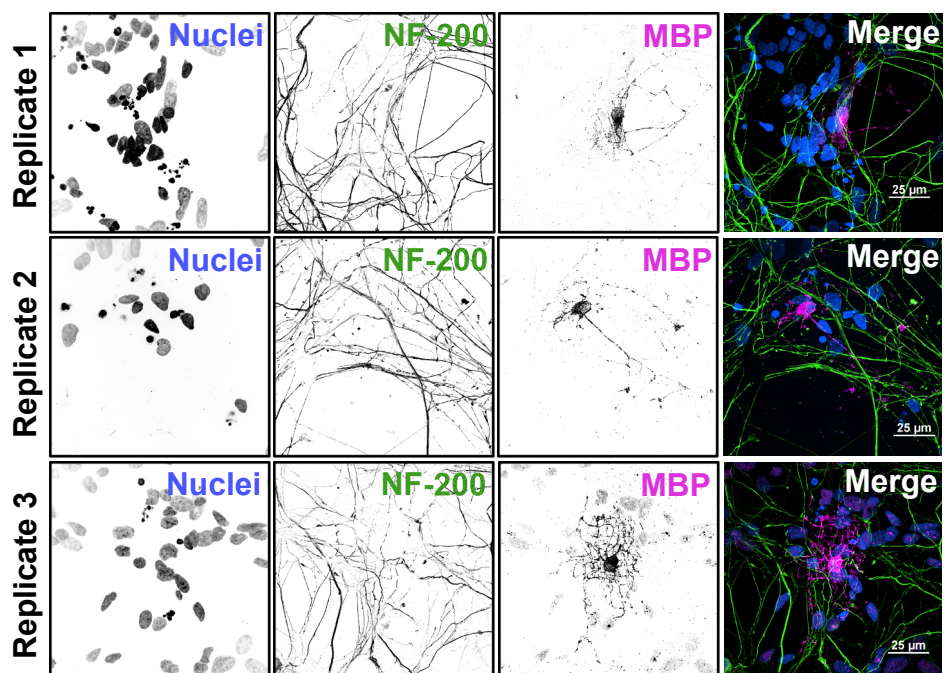

Supplement: Supplementary file 7 — Figure S7: Validation of motor neuron differentiation and representative images of co‐culture. (A) Representative spinning disk confocal maximum intensity projections of immunofluorescent staining for nuclei (cyan), ISL1/2 (magenta), and βIII‐tubulin (green) in motor neurons derived from human induced pluripotent stem cells (scale bar = 25 μm). (B) Representative spinning disk confocal maximum intensity projections of immunofluorescent staining for nuclei (blue), Neurofilament‐200 (NF‐200) (green), and myelin basic protein (MBP) (magenta) in Day 90 co‐cultures with cells that were previously treated as OPCs with DMSO (vehicle) or S63845 (scale bar = 25 μm). [file GLIA-74-0-s009.pdf]

Supplementary Figure 8

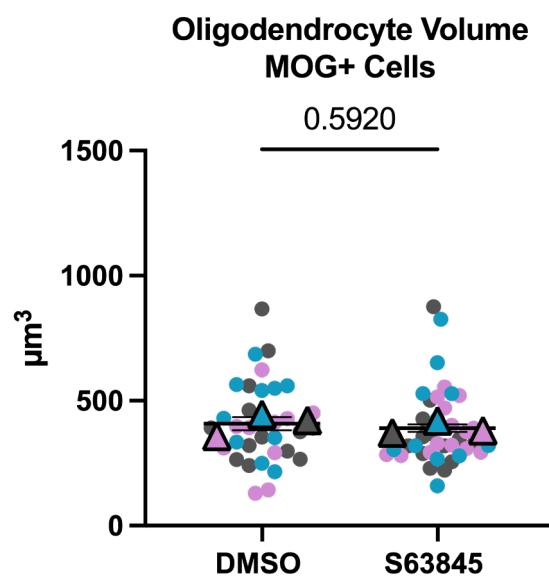

Supplement: Supplementary file 8 — Figure S8: Volume of MOG‐positive cells. Volume of myelin oligodendrocyte glycoprotein (MOG) positive cells in Day 90 co‐cultures with cells that were previously treated as OPCs with DMSO (vehicle) and S63845. Each color represents a biological replicate (n = 3), each dot represents a cell (10–15 per n), each triangle represents mean of biological replicate, analyzed by student's t‐test, error bars represent mean ± SEM. Conditions were blinded to experimenter for 3D reconstructions. [file GLIA-74-0-s007.pdf]

# Supplementary Figure 9

**A**

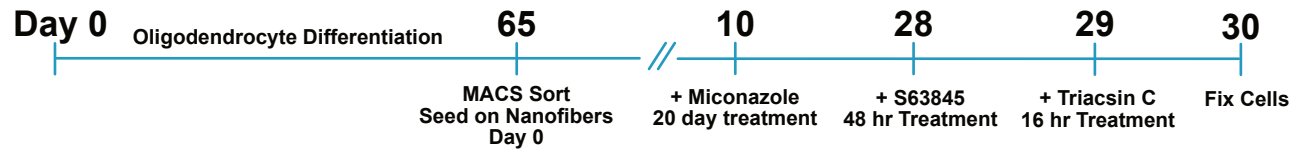

**B**

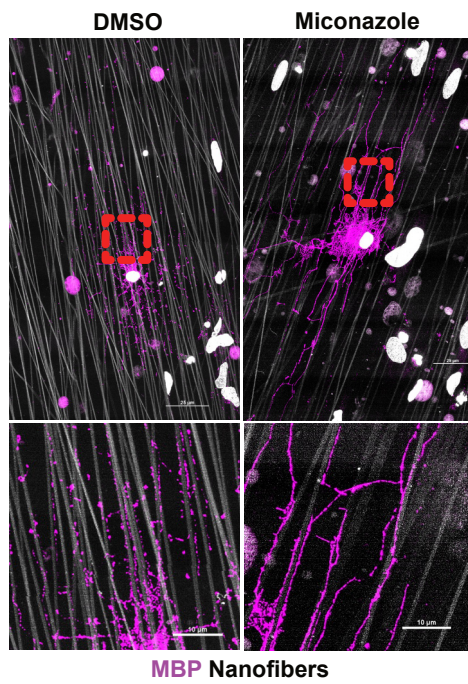

**C**

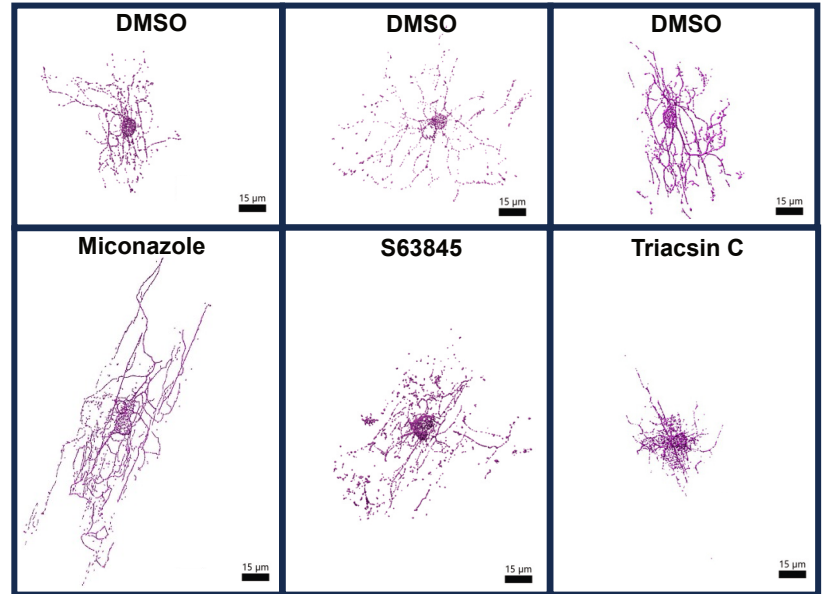

**D**

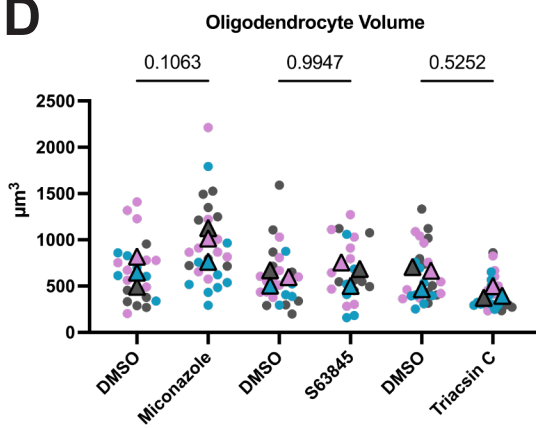

**E**

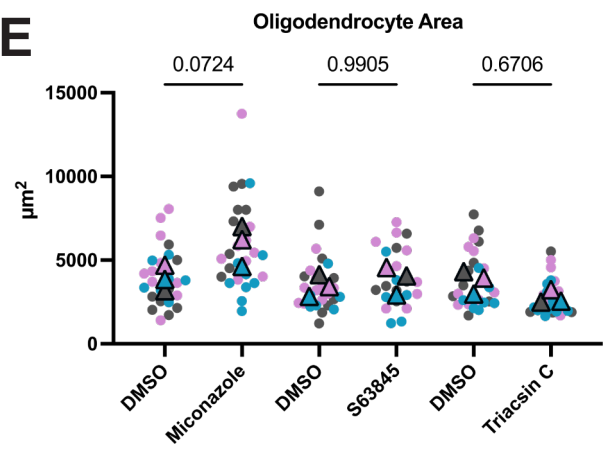

Supplement: Supplementary file 9 — Figure S9: Using a neuron‐free nanofiber assay to evaluate pharmacological inhibition of MCL‐1 and ACSL1. (A) Timeline of oligodendrocyte differentiation, seeding on nanofibers, treatment with miconazole, S63845, and Triascin C. At Day 65 of differentiation, cells undergo cell sorting using the MACS cell separation system. Cells were directly seeded onto nanofibers after MACS sorting, commencing nanofiber culture Day 0. At Day 10, a first set of cells were treated with miconazole or DMSO (vehicle) and treatment continued for 20 days until collection. A second set of cells were treated with S63845 or DMSO (vehicle) for 48 h at Day 28. At Day 29, third set of cells were treated with Triacsin C or DMSO (vehicle) for 16 h. Cells were fixed at Day 30 following treatments. (B) Representative SoRa maximum intensity projections of immunofluorescent staining for nanofibers (white) and MBP (magenta) in oligodendrocytes treated with DMSO (vehicle) and miconazole (scale bar = 25 μm). Zoom‐in of oligodendrocyte process on nanofiber (scale bar = 10 μm). (C) 3D reconstructions of MBP‐positive oligodendrocytes cultured on nanofibers with miconazole, S63845, Triacsin C, and their respective DMSO vehicle controls (scale bar = 15 μm). (D) Quantification of MBP‐positive oligodendrocyte volume in miconazole, S63845, Triacsin C, and respective DMSO (vehicle) treated cells. Each color represents a biological replicate (n = 3), each dot represents a cell (6–11 per n), each triangle represents the mean of biological replicate analyzed by ordinary one‐way ANOVA, error bars represent mean ± SEM. Conditions were blinded to the experimenter for 3D reconstructions. (E) Quantification of MBP‐positive oligodendrocyte area in Miconazole, S63845, Triacsin C, and respective DMSO (vehicle) treated cells. Each color represents a biological replicate (n = 3), each dot represents a cell (6–11 per n), each triangle represents the mean of biological replicate analyzed by ordinary one‐way ANOVA, error bars [file GLIA-74-0-s003.pdf]
