# Supplementary material for: A Human Model of Oligodendrocyte Development Shows MCL‐1 Influences Oligodendrocyte Morphogenesis
Source: Glia. 2025 Dec 19;74(2):e70128. doi: 10.1002/glia.70128 (PMC12717335; doi:10.1002/glia.70128)

Supplementary Figure 5

A

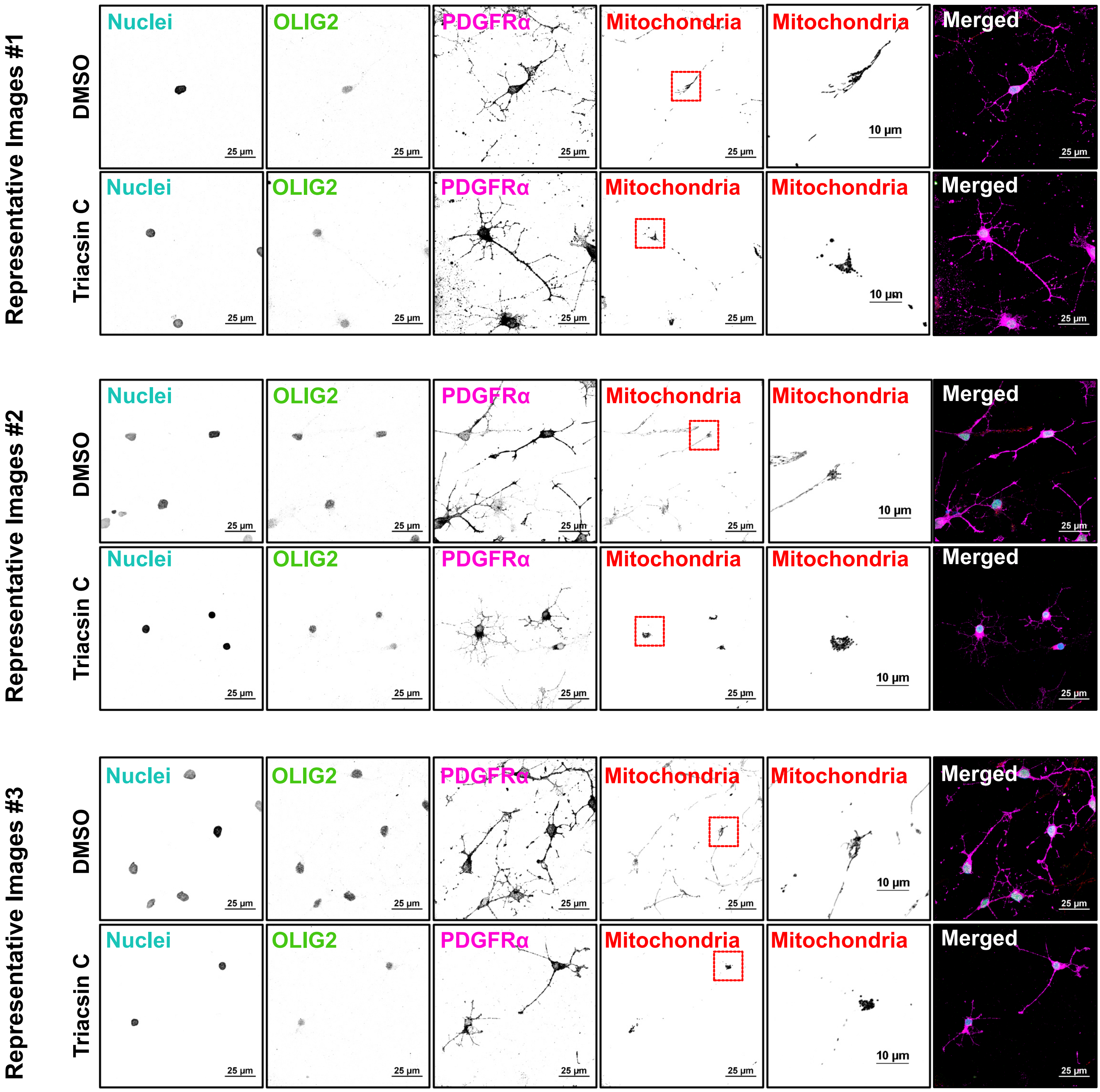

B

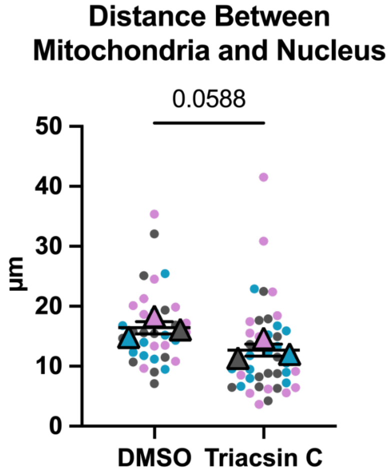

Supplement: Supplementary file 5 — Figure S5: Pharmacological inhibition of ACSL leads to robust alterations to mitochondrial morphology and localization. (A) Representative spinning disk confocal maximum intensity projections of immunofluorescent staining for nuclei (cyan), oligodendrocyte transcription factor 2 (OLIG2) (green), platelet‐derived growth factor receptor alpha (PDGFRα) (magenta), and mitochondria (red) in DMSO (vehicle) and Triacsin C treated OPCs (scale bar = 25 μm zoom in of mitochondria at the nucleus [scale bar = 10 μm]). (B) Quantification of the distance between mitochondria and nucleus. Each color represents a biological replicate (n = 3), each dot represents a cell (10–15 per n), each triangle represents mean of biological replicate, analyzed by student's t‐test, error bars represent mean ± SEM. [file GLIA-74-0-s004.pdf]
