# Supplementary material for: A Human Model of Oligodendrocyte Development Shows MCL‐1 Influences Oligodendrocyte Morphogenesis
Source: Glia. 2025 Dec 19;74(2):e70128. doi: 10.1002/glia.70128 (PMC12717335; doi:10.1002/glia.70128)
Supplement: Supplementary file 10 — Table S1: Detailed statistical report. [file GLIA-74-0-s005.docx]

**Supplementary Table 1: Detailed statistical report**

| Figure | Normality test  (if applicable) | Passed normality test  (alpha=0.05)?  (if applicable) | Statistical test | P value | t, df  (if applicable) | F, DFn, DFd  (if applicable) | Multiple Comparisons test  (if applicable) | Comparison, P value (if applicable) |
| --- | --- | --- | --- | --- | --- | --- | --- | --- |
| Figure 1B | N/A | N/A | Welch’s t test | 0.1120 | t=2.730, df=2 | N/A | N/A | N/A |
| Figure 1C | N/A | N/A | Welch’s t test | 0.0598 | t=3.903, df=2 | N/A | N/A | N/A |
| Figure 2B | Shapiro-Wilk test | Pass | Welch’s t test | 0.8990 | t=0.1353, df=3.970 | N/A | N/A | N/A |
| Figure 2C | Shapiro-Wilk test | Pass | Welch’s t test | 0.9336 | t=0.08894, df=3.848 | N/A | N/A | N/A |
| Figure 2D | Shapiro-Wilk test | Pass | Welch’s t test | 0.6203 | t=0.5364, df=3.978 | N/A | N/A | N/A |
| Figure 2E | Shapiro-Wilk test | Pass | Welch’s t test | 0.6736 | t=0.4562, df=3.720 | N/A | N/A | N/A |
| Figure 2F | Shapiro-Wilk test | Pass | Welch’s t test | 0.6425 | t=0.5069, df=3.496 | N/A | N/A | N/A |
| Figure 2H | Shapiro-Wilk test | Pass | Ordinary one-way ANOVA | 0.9050 | N/A | 0.1755 (3, 8) | Tukey's multiple comparisons test | DMSO Soma vs. DMSO Processes: 0.9810;  DMSO Soma vs. S63845 Soma: 0.8815;  DMSO Processes vs. S63845 Processes: 0.9996:  S63845 Soma vs. S63845 Processes: 0.9666 |
| Figure 3B | N/A | N/A | Welch’s t test | 0.9808 | t=0.02721, df=2 | N/A | N/A | N/A |
| Figure 3C | N/A | N/A | Welch’s t test | 0.1736 | t=2.076, df=2 | N/A | N/A | N/A |
| Figure 3D | N/A | N/A | Welch’s t test | 0.7745 | t=0.3273, df=2 | N/A | N/A | N/A |
| Figure 3E | N/A | N/A | Welch’s t test | 0.0632 | t=3.786, df=2 | N/A | N/A | N/A |
| Figure 4B | N/A | N/A | 2-way ANOVA | Interaction P value: 0.2637;  Row Factor P value: 0.2641;  Column Factor P value: 0.0005 | DMSO:BSA vs. DMSO:Palmitate t: 3.131;  DMSO:Palmitate vs. Etomoxir:Palmitate t: 1.699;  Etomoxir:BSA vs. Etomoxir:Palmitate t: 4.831 | DMSO:BSA vs. DMSO:Palmitate DF: 8;  DMSO:Palmitate vs. Etomoxir:Palmitate DF: 8;  Etomoxir:BSA vs. Etomoxir:Palmitate DF: 8 | Uncorrected Fisher's LSD | DMSO:BSA vs. DMSO:Palmitate: 0.0140;  DMSO:Palmitate vs. Etomoxir:Palmitate: 0.1277;  Etomoxir:BSA vs. Etomoxir:Palmitate: 0.0013; |
| Figure 4C | Shapiro-Wilk test | Pass | Welch’s t test | 0.0684 | t=2.709, df=3.193 | N/A | N/A | N/A |
| Figure 4E | N/A | N/A | 2-way ANOVA | Interaction P value:  0.5139; Row Factor P value:  0.8735; Column Factor P Value:  0.0010 | DMSO:BSA vs. DMSO:Palmitate t: 3.061;  DMSO:Palmitate vs. S63845:Palmitate t: 0.5991;  S63845:BSA vs. S63845:Palmitate t: 4.027 | DMSO:BSA vs. DMSO:Palmitate DF: 8;  DMSO:Palmitate vs. S63845:Palmitate DF: 8;  S63845:BSA vs. S63845:Palmitate DF: 8 | Uncorrected Fisher's LSD | DMSO:BSA vs. DMSO:Palmitate: 0.0156;  DMSO:Palmitate vs. S63845:Palmitate: 0.5656;  S63845:BSA vs. S63845:Palmitate: 0.0038 |
| Figure 4F | Shapiro-Wilk test | Pass | Welch’s t test | 0.1976 | t=1.694, df=2.733 | N/A | N/A | N/A |
| Figure 4H | N/A | N/A | 2-way ANOVA | Interaction P value:  0.0059; Row Factor P value:  0.0072; Column Factor P Value:  0.0066 | DMSO:BSA vs. DMSO:Palmitate t: 5.205;  DMSO:Palmitate vs. Triacsin C:Palmitate t: 5.163;  Triacsin C:BSA vs. Triacsin C:Palmitate t: 0.05905 | DMSO:BSA vs. DMSO:Palmitate DF: 8;  DMSO:Palmitate vs. Triacsin C:Palmitate DF: 8;  Triacsin C:BSA vs. Triacsin C:Palmitate DF: 8 | Uncorrected Fisher's LSD | DMSO:BSA vs. DMSO:Palmitate: 0.0008;  DMSO:Palmitate vs. Triacsin C:Palmitate: 0.0009;  Triacsin C:BSA vs. Triacsin C:Palmitate: 0.9544 |
| Figure 4I | Shapiro-Wilk test | Pass | Welch’s t test | 0.0200 | t=6.725, df=2.051 | N/A | N/A | N/A |
| Figure 5A | N/A | N/A | Welch’s t test | 0.3257 | t=1.291, df=2 | N/A | N/A | N/A |
| Figure 5B | N/A | N/A | Welch’s t test | 0.0881 | t=3.143, df=2 | N/A | N/A | N/A |
| Figure 5C | N/A | N/A | Welch’s t test | 0.9687 | t=0.04424, df=2 | N/A | N/A | N/A |
| Figure 5D | N/A | N/A | Welch’s t test | 0.0007 | t=37.36, df=2 | N/A | N/A | N/A |
| Figure 6C | Shapiro-Wilk test | Pass | Welch’s t test | 0.1989 | t=1.798, df=2.267 | N/A | N/A | N/A |
| Figure 6D | Shapiro-Wilk test | Pass | Welch’s t test | 0.2273 | t=1.620, df=2.367 | N/A | N/A | N/A |
| Figure 6E | Shapiro-Wilk test | Pass | Welch’s t test | 0.1144 | t=2.624, df=2.092 | N/A | N/A | N/A |
| Figure 6F | Shapiro-Wilk test | Pass | Welch’s t test | 0.1303 | t=2.101, df=2.877 | N/A | N/A | N/A |
| Figure 6G | Shapiro-Wilk test | Pass | Welch’s t test | 0.0240 | t=3.877, df=3.415 | N/A | N/A | N/A |
| Figure 6I | Shapiro-Wilk test | Pass | Ordinary one-way ANOVA | 0.1216 | N/A | 0.9912 (3, 8) | Tukey's multiple comparisons test | DMSO Soma vs. DMSO Processes: 0.1811;  DMSO Soma vs. S63845 Soma: 0.5309;  DMSO Processes vs. S63845 Processes: 0.9933:  S63845 Soma vs. S63845 Processes: 0.6769 |
| Figure 7B | Shapiro-Wilk test | Pass | Welch’s t test | 0.0179 | t=4.122, df=3.617 | N/A | N/A | N/A |
| Figure 7C | Shapiro-Wilk test | Pass | Welch’s t test | 0.1123 | t=2.713, df=2.017 | N/A | N/A | N/A |
| Figure 7D | Shapiro-Wilk test | Pass | Welch’s t test | 0.7210 | t=0.3859, df=3.649 | N/A | N/A | N/A |
| Figure 7E | Shapiro-Wilk test | Pass | Welch’s t test | 0.7021 | t=0.4137, df=3.672 | N/A | N/A | N/A |
| Figure 7G | N/A | N/A | Multiple paired t tests | 30 µm:0.054907;  40 µm:0.057008;  50 µm:0.018402 | N/A | N/A | N/A | N/A |
| Supplementary Figure 3 | Shapiro-Wilk test | Pass | Welch’s t test | 0.2426 | t=1.378, df=3.875 | N/A | N/A | N/A |
| Supplementary Figure 4A | N/A | N/A | 2-way ANOVA | Interaction P value: 0.0301;  Row Factor P value: 0.0219;  Column Factor P value: 0.0005 | DMSO:BSA vs. DMSO:Palmitate t: 2.090;  DMSO:Palmitate vs. Etomoxir:Palmitate t: 3.867;  Etomoxir:BSA vs. Etomoxir:Palmitate t: 5.811 | DMSO:BSA vs. DMSO:Palmitate DF: 8;  DMSO:Palmitate vs. Etomoxir:Palmitate DF: 8;  Etomoxir:BSA vs. Etomoxir:Palmitate DF: 8 | Uncorrected Fisher's LSD | DMSO:BSA vs. DMSO:Palmitate: 0.0700;  DMSO:Palmitate vs. Etomoxir:Palmitate: 0.0048;  Etomoxir:BSA vs. Etomoxir:Palmitate: 0.0004 |
| Supplementary Figure 4B | N/A | N/A | 2-way ANOVA | Interaction P value: 0.2245;  Row Factor P value: 0.6871;  Column Factor P value: 0.4984 | DMSO:BSA vs. DMSO:Palmitate t: 1.432;  DMSO:Palmitate vs. Etomoxir:Palmitate t: 0.6355;  Etomoxir:BSA vs. Etomoxir:Palmitate t: 0.4294 | DMSO:BSA vs. DMSO:Palmitate DF: 8;  DMSO:Palmitate vs. Etomoxir:Palmitate DF: 8;  Etomoxir:BSA vs. Etomoxir:Palmitate DF: 8 | Uncorrected Fisher's LSD | DMSO:BSA vs. DMSO:Palmitate: 0.1899;  DMSO:Palmitate vs. Etomoxir:Palmitate: 0.5428;  Etomoxir:BSA vs. Etomoxir:Palmitate: 0.6789 |
| Supplementary Figure 4C | N/A | N/A | 2-way ANOVA | Interaction P value: 0.5805;  Row Factor P value: 0.5525;  Column Factor P value: 0.0660 | DMSO:BSA vs. DMSO:Palmitate t: 1.098;  DMSO:Palmitate vs. S63845:Palmitate t: 0.8456;  S63845:BSA vs. S63845:Palmitate t: 1.912 | DMSO:BSA vs. DMSO:Palmitate DF: 8;  DMSO:Palmitate vs. S63845:Palmitate DF: 8;  S63845:BSA vs. S63845:Palmitate DF: 8 | Uncorrected Fisher's LSD | DMSO:BSA vs. DMSO:Palmitate: 0.3043;  DMSO:Palmitate vs. S63845:Palmitate: 0.4223;  S63845:BSA vs. S63845:Palmitate: 0.0923 |
| Supplementary Figure 4D | N/A | N/A | 2-way ANOVA | Row Factor P value: 0.7595;  Column Factor P value: 0.4014 | DMSO:BSA vs. DMSO:Palmitate t: 0.8806;  DMSO:Palmitate vs. S63845:Palmitate t: 0.3156;  S63845:BSA vs. S63845:Palmitate t: 0.8806 | DMSO:BSA vs. DMSO:Palmitate DF: 8;  DMSO:Palmitate vs. S63845:Palmitate DF: 8;  S63845:BSA vs. S63845:Palmitate DF: 8 | Uncorrected Fisher's LSD | DMSO:BSA vs. DMSO:Palmitate: 0.4014;  DMSO:Palmitate vs. S63845:Palmitate: 0.7595;  S63845:BSA vs. S63845:Palmitate: 0.4014 |
| Supplementary Figure 4E | N/A | N/A | 2-way ANOVA | Row Factor P value: 0.2955;  Column Factor P value: 0.1680 | DMSO:BSA vs. DMSO:Palmitate t: 1.499;  DMSO:Palmitate vs. Triacsin C:Palmitate t: 1.111;  Triacsin C:BSA vs. Triacsin C:Palmitate t: 1.499 | DMSO:BSA vs. DMSO:Palmitate DF: 8;  DMSO:Palmitate vs. Triacsin C:Palmitate DF: 8;  Triacsin C:BSA vs. Triacsin C:Palmitate DF: 8 | Uncorrected Fisher's LSD | DMSO:BSA vs. DMSO:Palmitate: 0.1680;  DMSO:Palmitate vs. Triacsin C:Palmitate: 0.2955;  Triacsin C:BSA vs. Triacsin C:Palmitate: 0.1680 |
| Supplementary Figure 4F | N/A | N/A | 2-way ANOVA | Row Factor P value: 0.2696;  Column Factor P value: 0.8457 | DMSO:BSA vs. DMSO:Palmitate t: 0.2002;  DMSO:Palmitate vs. Triacsin C:Palmitate t: 1.177;  Triacsin C:BSA vs. Triacsin C:Palmitate t: 0.2002 | DMSO:BSA vs. DMSO:Palmitate DF: 8;  DMSO:Palmitate vs. Triacsin C:Palmitate DF: 8;  Triacsin C:BSA vs. Triacsin C:Palmitate DF: 8 | Uncorrected Fisher's LSD | DMSO:BSA vs. DMSO:Palmitate: 0.8457;  DMSO:Palmitate vs. Triacsin C:Palmitate: 0.2696;  Triacsin C:BSA vs. Triacsin C:Palmitate: 0.8457 |
| Supplementary Figure 5B | Shapiro-Wilk test | Pass | Welch’s t test | 0.0588 | t=2.621, df=3.999 | N/A | N/A | N/A |
| Supplementary Figure 6A | N/A | N/A | Welch’s t test | 0.5187 | t=0.7766, df=2 | N/A | N/A | N/A |
| Supplementary Figure 6B | N/A | N/A | Welch’s t test | 0.7749 | t=0.3268, df=2 | N/A | N/A | N/A |
| Supplementary Figure 8 | Shapiro-Wilk test | Pass | Welch’s t test | 0.5920 | t=0.5954, df=3.121 | N/A | N/A | N/A |
| Supplementary Figure 9D | Shapiro-Wilk test | Pass | Ordinary one-way ANOVA | 0.0083 | N/A | 0.3280 (5, 12) | Tukey's multiple comparisons test | DMSO vs. Miconazole: 0.1063;  DMSO vs. S63845: 0.9947;  DMSO vs. Triacsin C: 0.5252 |
| Supplementary Figure 9E | Shapiro-Wilk test | Pass | Ordinary one-way ANOVA | 0.0080 | N/A | 0.3427 (5, 12) | Tukey's multiple comparisons test | DMSO vs. Miconazole: 0.0724;  DMSO vs. S63845: 0.9905;  DMSO vs. Triacsin C: 0.6706 |
